# Fundamental Concepts and Evolution of Wi-Fi User Localization: An Overview Based on Different Case Studies

**DOI:** 10.3390/s20185121

**Published:** 2020-09-08

**Authors:** Guenther Retscher

**Affiliations:** Department of Geodesy and Geoinformation, TU Wien, 1040 Vienna, Austria; guenther.retscher@tuwien.ac.at; Tel.: +43-1-58801-12847

**Keywords:** received signal strength RSS, round trip time RTT measurements, location fingerprinting, lateration, combination and fusion of techniques, positioning algorithms, indoor smartphone user localization

## Abstract

Indoor positioning poses a number of challenges, especially in large and complex buildings. Several effects, such as signal attenuation, signal fluctuations, interference, and multipath play a decisive role in signal propagation. The severity of each challenge depends on the method and technology adopted to perform user localization. Wi-Fi is a popular method because of its ubiquity with already available public and private infrastructure in many environments and the ability for mobile clients, such as smartphones, to receive these signals. In this contribution, the fundamental concepts and basics and the evolution of Wi-Fi as the most widely used indoor positioning technology are reviewed and demonstrated using four different conducted case studies. Starting from an analysis of the properties of Wi-Fi signals and their propagation, suitable techniques are identified. The mathematical models of location fingerprinting and lateration are consolidated and assessed as well as new technology directions and developments highlighted. Results of the case studies demonstrate the capability of Wi-Fi for continuous user localization also in dynamic environments and kinematic mode where the user walks with a usual step speed. However, to achieve acceptable localization accuracy, calibration of the devices is required to mitigate the variance problems due to the device heterogeneity.

## 1. Introduction

Location is one of the most important contexts for computing devices, especially mobile devices such as smartphones. These devices have evolved into promising computing platforms including communication capability, network access, and multi-function embedded sensors. With the rapid development of mobile Internet, getting accurate location information becomes more and more significant. Location information can be used to provide various Location-Based Services (LBS) (see e.g., [1,2,3,4]). Localization in GNSS (Global Navigation Satellite Systems) denied/challenged indoor/outdoor and transitional environments represents a challenging research problem. Many different types of Indoor Positioning System (IPS) using radio frequencies for absolute user localization are developed based either on the deployment of special hardware in the indoor environment or the use of already available signals, the so-called signals-of-opportunities [5]. Wi-Fi is one of the most widely used signal-of-opportunity for positioning and tracking mobile users, as it is commonly adopted for smartphone-based indoor positioning systems due to the availability of already deployed infrastructure for communications and the ability for mobile devices to receive these signals. Nowadays, a high number of Access Points (APs) of public and private networks can be sensed providing a high signal ubiquity.

The vast majority of current IPS are designed for sub-meter accuracy in position estimation, which is unnecessary for certain indoor navigation applications, such as most LBS as well as pedestrian navigation (see e.g., [6]). Then, a room-level or region-level granularity of location is sufficient [7,8,9,10]. The use of Wi-Fi is predestinated and capable of achieving such a level of precision with high performance. Therefore, Wi-Fi signals have a high potential to employ them for numerous applications for localization and guidance. Thus, Wi-Fi advances in wireless communication and the consequent ubiquity of Wi-Fi infrastructure provide the ability to extract human-related information, such as location, movement, and other activity by analyzing wireless connectivity between mobile clients and the APs [11].

Localization using Wi-Fi is either based on direct measurements of the Received Signal Strengths (RSS) of the surrounding Wi-Fi APs or on the measurement of the two-way travel time, i.e., the Round Trip Time (RTT), between the mobile device and several APs. Therefore, localization methods include lateration and fingerprinting algorithms. Thereby, the RSS-based fingerprinting approach has the advantage that no direct line-of-sight (LoS) is required and it does not need any prior knowledge of the APs deployment and their location [12]. It works also well in environments with high multipath. Fingerprinting is a so-called feature-based technology as a spatial variable feature, the RSS, is measured and georeferenced. Thereby, the measured RSS are used directly for a matching process where the current RSS measurements in the positioning phase are matched to previously measured RSS in a preceding system training phase. Fingerprinting is not so severely affected by signal fluctuations and interference than RSS-based lateration methods. This is why Wi-Fi fingerprinting is currently the most popular technology for an IPS. On the other hand, new developments for lateration-based approaches lead to further possibilities in Wi-Fi positioning. By measuring the RTT [13,14], the double distance between the AP and the mobile client can be obtained, which usually can provide higher accuracies for the ranges than with RSS-based methods. In RSS-based ranging [15], complex signal propagation models have to be derived and employed for the estimation of the signal path loss. The way to go in the future is a combination of different techniques to be able to use the advantages of all individual localization approaches.

This contribution reviews the major developments in the field of Wi-Fi user localization verified by investigations and results from different case studies. The remainder of the paper is structured as follows: In Section 2, first, the basics and theoretical foundations and major challenges in Wi-Fi positioning are discussed, including negative effects of Wi-Fi signal propagation due to heavy signal fluctuations. Section 3 is the main section of the paper presenting the mathematical models for the most commonly employed approaches ranging from location fingerprinting to RSS- and RTT-based lateration. The four case studies described in Section 4 verify the current state-of-the-art in Wi-Fi localization. In Section 5, the evolution of Wi-Fi positioning starting from its beginnings is briefly summarized and new developments are discussed. Finally, the paper is concluded and outlook on future work at TU Wien is given in Section 6.

## 2. Basics and Challenges in Wi-Fi Localization

In this section, the principle of operation of a Wi-Fi network is briefly reviewed. Then, a special emphasis is led on the full discussion of the challenges in Wi-Fi user localization. Influences on the results are caused by signal damping, interference, fluctuations and noise, shielding due to the human body of the user, dependency on the type of mobile device, duration of a single RSS signal scan, and other challenges.

### 2.1. Principle of Operation of a Wi-Fi Network

Wi-Fi refers to a local wireless network, which is classified under the IEEE (Institute of Electrical and Electronics Engineers) standard 802.11. Over the years since its introduction, the 802.X standard has been further developed by several extensions, with each extension having its own characteristics, such as frequency band used or the range. However, the main objective of any extension is to increase the data rate, which does not play a role for an IPS. Table 1 summarizes the most important extensions with their characteristics. Some older smartphones still work with the 802.11a/b/g/n standard. The 802.11ac standard is integrated in most modern electronic devices. The standard 802.11ax (Wi-Fi 6) is a robust, highly efficient signal transmission for better operation at significantly lower signal strengths. The 802.11-2016 standard, also known as IEEE 802.11-REVmc, is a revision based on IEEE 802.11-2012 that includes five extensions (ae, aa, ad, ac, and af). With this standard, the FTM (Fine Time Measurement) protocol has been introduced, which allows the precise measurement of the signal Time-of-Flight (ToF) of two devices, enabling position determination by means of Wi-Fi RTT (Round Trip Time) (see Section 3.2.2). As shown in Table 1, the Wi-Fi signals are transmitted via the freely usable ISM band (Industrial, Scientific, and Medical Band) at 2.4 and 5 GHz. This corresponds to a wavelength of 12.5 and 6 cm, respectively. The frequency bands are used in channels with a bandwidth of 20 MHz in a 2.4 GHz band and 40 MHz in a 5 GHz band. The IEEE 802.11ac and 802.11.ax standards also include transmission bandwidths of 80 and 160 MHz, so that a more precise time-based position determination can be achieved with these Wi-Fi signals [16]. Thus, changes in signal transmission and improved utilization of the frequency bands were made. The optimal utilization of the frequency ranges is precisely defined in the individual standards. The TPC (Transmit Power Control) reduces the transmission power depending on the need. For example, if there is a good connection between the devices, the transmission power is reduced. With the help of DFS (Dynamic Frequency Selection), the AP recognizes other radio systems independently and can switch to a different frequency. This ensures that radar, satellite, and positioning services are not disturbed. The combination of TPC and DFS allows the APs to determine the channels with the best availability and to use the lowest possible transmission power. Therefore, the user only gets the transmission power that is required for the current distance to the AP.

Each frequency band has special advantages and disadvantages. Basically, the higher the frequency, the shorter the range (due to the higher signal attenuation). Thus, the 2.4 GHz band theoretically has a greater range, as it overcomes shielding materials with less loss. However, it has the disadvantage that the frequency band can be used with other electronic devices’ respective radio technologies, such as Bluetooth, microwave ovens, radio remote controls, etc. Therefore, it is more susceptible to interference. The advantage of the 5 GHz band is the significantly higher data transfer rate; however, it does not play a role in an IPS, as no data is transmitted, but only the signal strength is measured. The big disadvantage of the 5 GHz band is that the Wi-Fi signal is shielded more heavily from walls.

Two different operating modes can be used with Wi-Fi. In the ad-hoc mode, all parts of the network are equal, and communications takes place between the clients. The infrastructure mode is similar to the construction of a mobile network. An AP provides access to the wired network and handles the transfer of data to all the users. This is also called the Basic Service Set (BSS). The spatial coverage of an AP is called a radio cell, the size of which depends on the transmitting power and spatial conditions. Here, fluctuating influences, such as humidity in the air and the building fabric, play a major role. Ranges in the order of several kilometers are possible. Inside buildings, the signal usually has a shorter range (20 to 100 m) depending on the structure, but it can also pass through walls [17]. However, walls weaken the Wi-Fi RSS significantly, but surfaces can also serve as reflectors. The number of Wi-Fi users in a radio cell also has an influence on their size. As the number of users increases, the AP reacts by regulating the transmission power in order to provide all clients with information. The cell shrinks as a result. Opposite, the effect also occurs. A network with multiple APs (Extended Service Set, ESS) achieves greater spatial coverage. In Europe, the maximum output of an antenna is 20 dBm EIRP (Equivalent Isotropically Radiated Power). This means that no antenna can radiate more than an isotropic antenna into which 20 dBm are fed. The Wi-Fi networks are recognized via their SSID (Service Set Identifier), and the APs are identified via their BSSID (Basic Service Set Identifier) or via the MAC (Media Access Control) address. The MAC address is only assigned once worldwide, similar to a kind of a fixed serial number, and it is used for the unique detection of computer hardware. Each MAC address consists of 48 bits and is often represented as a 12-digit hexadecimal number. Then, the first six digits provide information about the manufacturer of the hardware, as each manufacturer is assigned certain MAC addresses by the IEEE. If a client in an ESS changes from one wireless cell to another, this is called roaming. An overlap between the cells is important because otherwise, there will be a disconnection. Furthermore, it is important that different channels are assigned to the APs, as otherwise, interference will occur [18]. Since the signal strengths, the SSID, and the associated MAC addresses can also be retrieved without an authenticated connection, this information is freely available. Thus, Wi-Fi positioning can be carried out autonomously, avoiding privacy concerns that typically occur with other positioning technologies [19].

An AP transmits small data packets (so-called beacons) in about every 100 ms, which contain the SSID and MAC address. This ensures continues data transmission. The mobile device receives the signal and can identify the AP using the MAC address. Therefore, various smartphone applications can record the signal strength of the surrounding APs. The transmission power *P*, which is emitted by an AP, is specified in the logarithmic unit decibel-milliwatt (dBm). The unit Bel is a logarithmic quantity and is defined by reference to a certain value. In the case of dBm, the reference value is 1 milliwatt (mW). One mW corresponds to 0 dBm; values above 1 mW result in positive dBm values, values below 1 mW result in negative dBm values. The functional relationship is given by:

(1) $$P\left[\mathrm{dBm}\right]=10\cdot log_{10}\left(\frac{P\left[\mathrm{mW}\right]}{1\left[\mathrm{mW}\right]}\right).$$

The transmission power of an AP depends, among other things, on the frequency band on which the signal is transmitted. With the 2.4 GHz band, the legally regulated maximum transmission power is 100 mW, which corresponds to 20 dBm. In the 5 GHz band, the maximum transmission power in a building is 200 mW, which respectively corresponds to approximately 23 dBm. An AP always transmits with the transmission power determined by the TPC. Due to the signal attenuation, the RSS decreases along the propagation, so that RSS values between −20 and 100 dBm are usually measured at the receiver. This relationship is what is used in the signal strength measurement, if the range is to be derived. That is why usually, logarithmic path loss models are employed for a conversion of the RSS into a range to the AP (see Section 3.2.1).

### 2.2. Challenges for Localization

This section presents some challenges that may arise when a signal is transmitted from the transmitter to the receiver. The severity of each challenge thereby depends on the method and technology used to determine a position fix. The challenges for positioning described in the following often occur in a localization system based on Wi-Fi. Due to signal damping and attenuation as well as signal fluctuations and noise, Wi-Fi positioning is normally not robust against dynamic changes in the environment.

#### 2.2.1. Signal Damping

**Figure 1:**
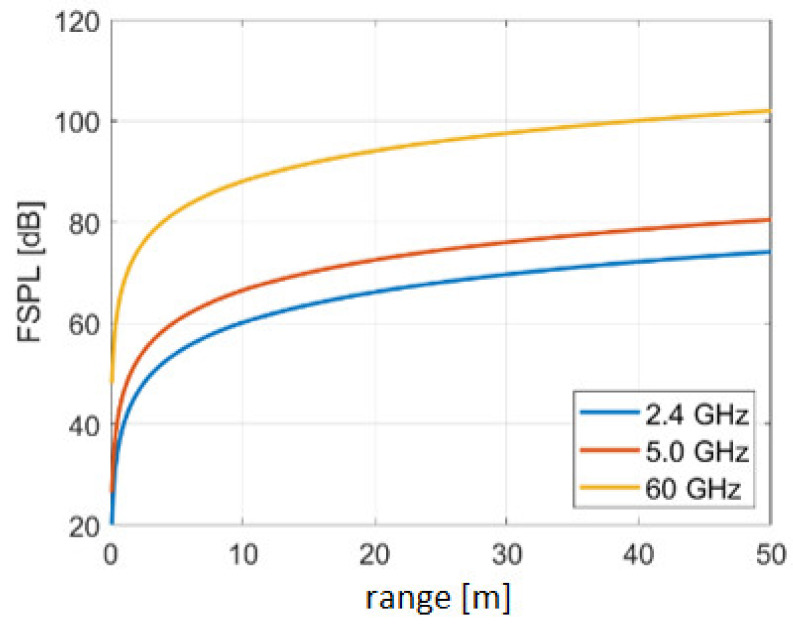
Free space path loss (FSPL) in dependence on the range for three different frequencies.

A signal loses energy in the course of propagation and is thus weakened. This attenuation is called damping in free space *FSPL* (Free Space Path Loss) and describes the reduction of the power density of an electromagnetic wave in free space, i.e., without interference from damping media, such as air or interference caused by reflections. The attenuation thereby depends on the signal frequency. If the signal weakens with increasing distance from the transmitter, the signal-to-noise ratio also decreases. The damping is usually given in decibels (dB), i.e., on a logarithmic scale, and it is described by means of the Friis transference equation. It has the following form for the emerging damping on dependence of the distance and wavelength under ideal theoretical conditions [20]:

(2) $$FSPL\;\left[\mathrm{dB}\right]=10\cdot {log}_{10}{\left(\frac{4\cdot \pi \cdot d\cdot f}{c}\right)}^{2}$$

where d is the distance between the transmitter and receiver in [m], f is the frequency of the signals in [Hz], and c is the propagation speed in [m/s^2^]. From the equation, it can be seen that the power decreases with the square of the distance to the transmitter. This theoretical connection is valid for direct LoS between the transmitter and receiver. The antenna gain is thereby excluded. In practice, an empiric logarithmic distance model can be derived from Equation (2), because also with LoS signals, reflections and damping due to physical objects occur. This empiric propagation model is described in Section 3.2.1 in more detail. Figure 1 shows the damping in free space *FSPL* for different frequencies. It can be seen that the signal is already considerably attenuated within a few meters from the transmitter and that the attenuation increases with the increasing frequency. The damping attenuation increases with distance and frequency, so that the signal is attenuated along the direction of propagation.

The influence of temperature, atmospheric pressure, and air humidity plays only a low role in the propagation of Wi-Fi signals in indoor environments, because on the one hand, short signal paths usually happen; on the other hand, damping effects are by far a bigger disturbance influence. Furthermore, by the propagation of Wi-Fi signal, the waves’ absorption and reflection effects play an essential role and have a large influence on the RSS. The following effects occur above all while the signal crosses between two different media [15]:

- Absorption: change of the energy in another form (e.g., in warmth);
- Diffraction: change of the propagation direction of the waves by an edge or a narrow gap;
- Refraction: change of the propagation direction;
- Reflection: tossing of the wave mainly from smooth surfaces; and
- Dispersion: tossing of the wave in different directions and strengths mainly from rough surfaces.

For an RSS to range conversion, not only does it matter that the ray path extends in contrary to a straight path between the transmitter and receiver due to diffraction and refraction, but also the damping of the signal makes by far a larger interference in the result of the range calculation due to absorption and dispersion. Table 2 summarizes the damping values of different materials relevant to signal propagation inside buildings for the 2.4 GHz frequency band. The extent of attenuation depends on the material properties, such as the kind of the building fabric and structure, e.g., caused by the construction type and the geometry of the medium. For instance, concrete reinforcement results in a higher damping value than that of a wall made out of bricks [21].

Two simple propagation models are commonly employed, i.e., the one-slope and multi-wall model [15]. The first model is a very simple empiric model that is based on the principle on the free space loss of the signals. The damping depends thereby only on the logarithmic distance between the transmitter and receiver and the reference RSS. Contrary, the semi-empiric multi-wall model considers the damping characteristics of existing walls between the transmitter and receiver along the direct path in the signal propagation estimation model. In simple environments, acceptable results can be achieved; however, the limitations of this model are soon reached if small structures, such as corridors, columns, narrow staircases, etc., and different materials in the walls exist. In Section 4.3, the resulting positioning accuracies using these two models in comparison with a newly developed differential approach are presented.

#### 2.2.2. Signal Interference

Another effect is the interference, which describes the change in amplitude when two or more waves are superimposed. The waves can either extinguish each other or amplify. This can result in either the signal strength being measured incorrectly or the signal not being measured at all. On the one hand, this can be by other devices, or on the other hand, it can also happen within an ESS if several APs are running unfavorably in the same frequency channel [20,21]. As Wi-Fi operates in license-free frequency domains, the Internet is an important part of our mobile society that has led to a widespread deployment of public and private APs. Nevertheless, it comes along with an increase of disturbances and interference, which may affect the positioning with such wireless signals. Jumps can appear in the Wi-Fi positioning results, causing deviations for the location estimation when a Wi-Fi signal fluctuates under the condition of severe interference [22]. Besides, in practice, it must be considered that not only one effect can occur, but different effects can come in different orders (e.g., ray beam guidance in narrow corridors).

#### 2.2.3. Signal Fluctuations and Noise

**Figure 2:**
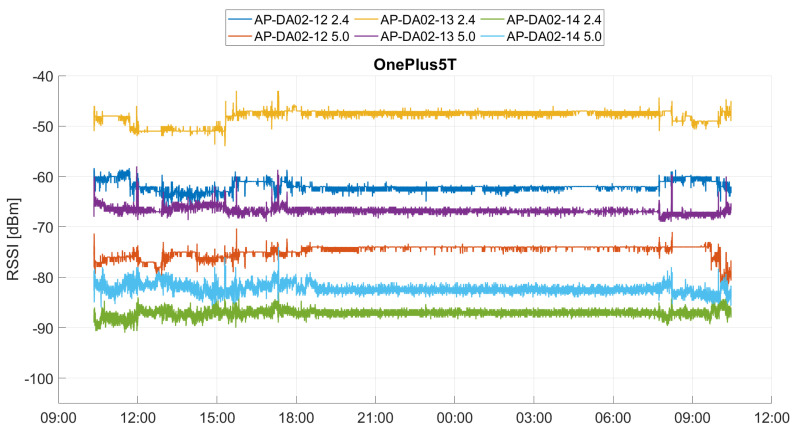
Signal strengths time series of long-term recordings with a smartphone to three Access Points (APs) for the 2.4 and 5 GHz frequency bands.

Each signal is subject to spatial and temporal fluctuations, which is why the RSS at the same location change over time. The fluctuations thereby depend on the technology and the physical environment (e.g., persons or objects) and can also be caused by external influences, such as temperature and humidity. In addition, some systems react to the number of receivers in the vicinity of the transmitter with a change in transmission power. Positioning by means of Wi-Fi location fingerprinting (see Section 3.1) depends, among other things, on the fluctuations and noise of the signal, and it is normally not robust against dynamic changes in the environment. Since a Wi-Fi signal is subject to natural fluctuations, the signal strength at the same location also changes over time. Furthermore, the multipath effect plays a major role, as the Wi-Fi signals are reflected and scattered due to walls, persons, and objects during propagation. If a signal is emitted, several signals always arrive at the receiver. Therefore, the RSS is a combination of the LoS signal and several multipath signals. However, the multipath effect has no influence on the Wi-Fi fingerprint if the environment remains the same. However, it may be due to short-term obstacles (people, opening or closing a door, etc.) that unexpected multipath effects that can amplify or weaken the signal. In addition, the AP can also be temporarily inaccessible or provide incorrect RSS. This is due to unexpected errors, interference, loss of energy, or even deliberate attacks by third parties. The signal fluctuations can also be caused by the dynamic transmission power of an AP. For instance, APs can dynamically deactivate the 2.4 GHz band and send it twice to the 5 GHz band instead. This decision is made by a central controller, which continuously outputs the optimal settings for the APs. The dynamic transmitting power depends on many factors, and a prediction is almost impossible. Using long-term measurements, these signal fluctuations can be analyzed. Figure 2 shows long-term RSS recordings over 24 h carried out with a smartphone in an office setting of three APs in close proximity to the smartphone. The APs have been broadcasted in both the 2.4 and 5 GHz band. Outliers larger than three times the mean absolute deviation from the median with a window length of one minute were removed from the data. Overall, fluctuations of ±5 dBm occur during the day where people are present in the building. At 19:00, the average standard deviation is 1.2 dB, after which the signals become much more stable with fluctuations of only ±1 dBm during the night. From 07:30, the fluctuations get larger again. This shows that the fewer people in the building, the more stable the signals, as the transmission power did not constantly change. Smartphones other than the one shown in Figure 2 yielded similar results at the same location.

In order to solve the problem of fluctuating transmission power, a differential approach introduced by the author [15] can be employed. For example, the fingerprinting radio map can consist of the relative relationships between pairs of APs instead of the absolute RSS values or the differences between the RSS values of an AP. If the current transmission power of all APs is available to the IPS in real time, the influence of the dynamic transmission power can be minimized by means of differentiation. A similar possibility would be to permanently monitor the signal strength by means of receivers at known locations (so-called reference stations) near the APs and then to calculate the difference to the reference value at each point in the radio map. Then, the radio map consists only of the differential values, and in the on-line positioning phase, the differential values are also calculated and then applied to correct the users’ location (see Section 3.2.3).

#### 2.2.4. Influence of the Human Body on RSS

**Figure 3:**
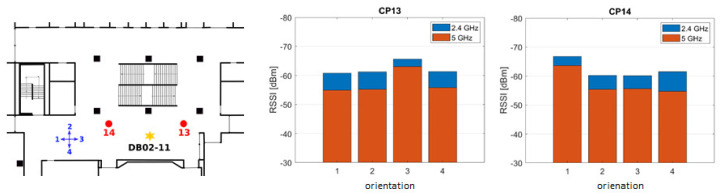
Influence of the human body on the orientation at two test locations CP 13 and CP 14.

Signal damping is also caused by the human body, because the human body consists of approximately of 70% water. Water has the resonance frequency of 2.4 GHz, which is equivalent to the Wi-Fi signals in the first frequency band. Not only the immediate nearness of the user to the smartphone plays a role, a far bigger problem is caused by the location of the user respectively to the location of an AP. Thus, the effect is different regarding the dependence of the orientation of the user, and it has to be accounted for if the RSS is observable. However, this damping effect is very difficult to be modeled, and it can be considered only an iterative positioning approach. Kaemarungsi and Krishnamurthy [20] mentioned values in signal fading of 9.32 dB for LoS and 5.81 dB for non LoS (NLoS). In a practical test, the RSSs were measured in four 90° offset orientations in order to investigate the signal attenuation of a human body. Figure 3 shows the RSSs in the four orientations between an AP (DB02-11) and a smartphone at two test points CP 13 and CP 14 inside a building if the smartphone is held by a person in front of the body at chest height. At both points, the signal strength was weaker measured in the orientation in which the body was located between the smartphone and the AP, i.e., for CP 13 in orientation 3 and for CP 14 in orientation 1, respectively. The signal attenuation by the human body lies between around 4 and 9 dBm; see Table 3. Thus, a significant weakening by the human body can be observed. It is interesting to note that the attenuation on the 5 GHz band is at 2 to 3 dBm stronger than on the 2.4 GHz band. Thus, in Wi-Fi location fingerprinting (see Section 3.1), training measurements are usually performed in four orientations.

#### 2.2.5. Device Dependence Due to Their Heterogeneity

**Figure 4:**
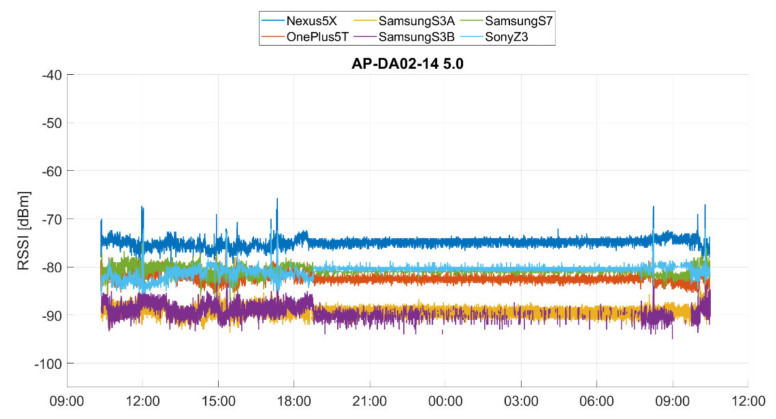
Signal strengths time series of long-term recordings with six smartphones of one AP.

Not only do the signal fluctuations make it difficult for localization, but also the observed RSS values pose a major challenge. Smartphones have different sensor properties, which is why the signal strengths are received differently at the same location. The reason for this is that different types of receivers (Wi-Fi chip-sets) and antennae are found in each smartphone, causing attenuation. These hardware differences negatively affect the accuracy of localization due to the usual use of heterogeneous devices. Luo and Zhan [23] reported on the impact of hardware on RSS recordings, showing that different hardware can result in significant variation of up to 25 dBm, even at the same time and place. In order to investigate the different reception sensitivity of different smartphones further, an analysis of the long-term observations was carried out. The device-dependent RSS of six smartphones from one AP are shown in Figure 4. Significant differences of up to 14 dBm for the devices can be seen; the RSSs thereby range from −75 to −89 dBm.

**Figure 5:**
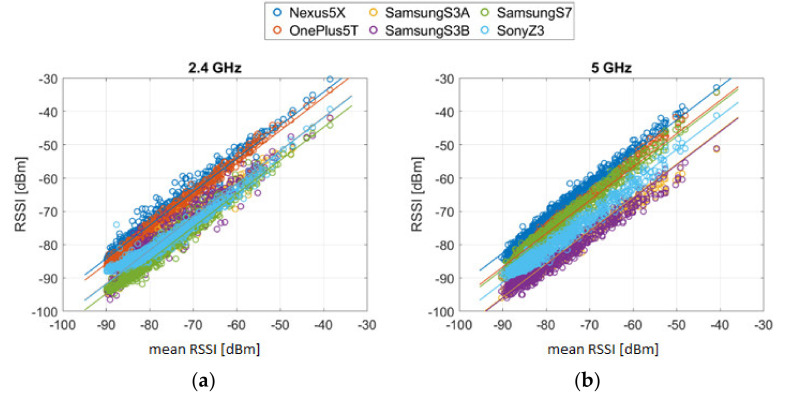
Multivariate linear regression for offset determination of individual smartphones in the system calibration of heterogeneous devices for (**a**) the 2.4 GHz and (**b**) 5 GHz frequency band.

**Figure 6:**
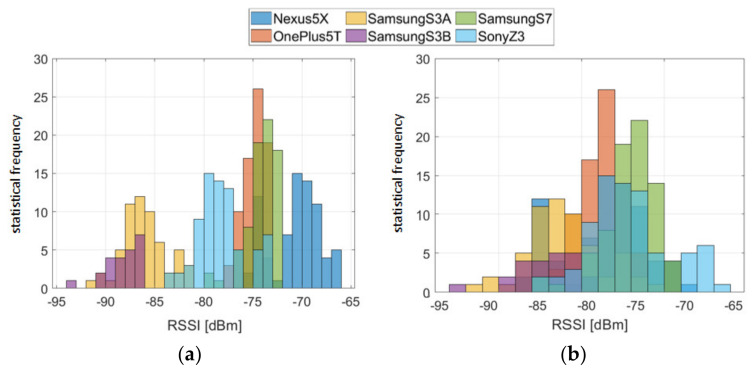
RSS values before (**a**) and after (**b**) the calibration.

If a method referred to as crowdsourcing (see e.g., [24,25,26,27,28]) is employed where any user can contribute to a built-up fingerprinting database in the training phase (see Section 3.2.1), usually different mobile devices are participating, leading to further challenges. Furthermore, in the online positioning phase, different smartphones are used than in system training as well. In order to enable crowdsourcing and to be able to compare the heterogeneous fingerprints of different smartphones, a calibration must therefore be carried out before the positioning phase. A suitable approach is to conduct device calibration using a data-fitting method that creates a linear transformation from the new device to the reference device [12]. Then, the adjusted RSSs can be combined as crowdsourcing training data. A way would be to determine an offset to the average RSSs for each smartphone, which is then added to the RSS values. One solution can be to estimate a multivariate linear regression. For this purpose, the average value of all the measured RSS of an AP at each reference point location can be first determined for each smartphone. For the reference vector of the regression, the values of all devices can be averaged. Only those APs that were observed exclusively with each smartphone are used. The linear regression model can have this simple form:

(3) $$y_{RSS}=a_{S}\cdot x_{S}+b_{S}$$

where $x_{S}$ is the measured RSS from the smartphone S which should be calibrated, and $y_{RSS}$ is the averaged reference vector calculated from all average RSS values that are estimated with the linear regression model. For the regression model $a_{S}=\mathrm{const}.$ is assumed, so that the gradient is equal for each smartphone; see Figure 5. The calibration coefficients $a_{S}$ and $b_{S}$ have been determined individually for each frequency band. It is particularly noticeable that there are large differences between the frequency bands on all three Samsung devices than on the other smartphones. Then, the RSSs adjusted by means of the calibration coefficients can be used for the fingerprinting database. In Figure 6, the measured RSS values of one AP on an indoor test location are compared before and after the calibration. It can be seen that the variation range could be reduced from 27 to 16 dBm using the calibration. Overall, the average standard deviation of all measurements could be reduced from 4.2 to 3.0 dBm.

#### 2.2.6. Dependence on Duration of a Wi-Fi RSS Scan

Especially in kinematic user localization, Retscher and Leb [29] have seen that the positioning accuracy depends a lot on the duration of a single Wi-Fi RSS scan. The authors found that the duration can significantly differ for different mobile devices. In their tests, the scan duration for a single measurement ranged from around 1.2 to even over 4.5 s on average. As a result, a lower number of measurements is available in kinematic positioning for smartphones with a long scan duration causing a lower level of positioning accuracies. Average deviations from the ground truth of more than 5 m occurred with long scan durations for trajectories where the pedestrian user walked along with a normal step speed. Two examples are given in Section 4.2.

#### 2.2.7. Other Challenges

As a result of the widespread use of Wi-Fi, hundreds of APs may be visible in the area where localization has to be performed. These are not promised to be stable, since many public and private APs exist. Thus, the increase in number of APs makes this environment more complex and uncontrollable, which brings several challenges [30]. The major challenges are as follows:

- Different types of APs may exist, such as multiple SSIDs where several networks at one physical AP are provided;
- Different hardware increases the difficulty of positioning algorithms, as they may receive different RSS readings from the same AP, even at the same position and time;
- Some APs might be temporarily unavailable due to various reasons; some may be replaced by new ones; and
- APs come and go; as time passes by, some APs disappear and new APs may emerge.

All these cases lead to the variation of the number of APs. When the environment is crowded with hundreds of APs, the variation may become more significant, which might increase the positioning errors. Furthermore, it will be difficult to compare and match the fingerprints collected by other devices. Chen et al. [30] have analyzed the performance in such conditions using five fingerprint-based algorithms. They could see that the performance in a real-world environment with hundreds of APs could vary significantly due to the number of APs, time variance, and different devices. Furthermore, they have shown that the number of APs could change significantly after a relatively long period—for example, after several months. Thus, a frequent re-calibration has to be carried out. Li et al. [12] dealt with the high-dimensional classification problem caused by the massive number of APs sensed as well as the missing data issue due to fact that every AP is not necessarily visible in every scanning cycle. Instead of ignoring the invisible APs, the authors employed the so-called expectation maximization (EM) algorithm for incomplete data parameter estimation. Here, the missing data are handled under the missing at random (MAR) assumption, which is an iterative process that finds the maximum likelihood estimation (MLE) of the parameters until they converge in the presence of missing data [31,32,33]. This approach significantly improved the performance of location fingerprinting compared to approaches where just a constant low RSS value than measurable, such as setting the RSS value to 101 dBm, is used to replace the missing data.

## 3. Common Mathematical Models

This section presents mainly the two most commonly employed techniques in Wi-Fi positioning, i.e., the location fingerprinting and lateration-based approaches. For fingerprinting, deterministic and probabilistic approaches, and for lateration, methods based on the measurement of the RSS and Time-of-Flight referred to as Round Trip Time (RTT) measurements are discussed in more detail. The section concludes with a proposal for the fusion of the methods based on an assessment of the results from four case studies presented in the following Section 4.

### 3.1. Location Fingerprinting

Fingerprinting can be referred to as a feature-based positioning method, which locates a mobile device through the geographical dependency between positions and signal observables. Thus, a spatially varying feature, such as the Received Signal Strength Indicator (RSSI), is measured and used directly for position estimation. In contrast to lateration (Section 3.2), fingerprinting uses signal attenuation and the multipath effect to determine the position. The fingerprints are in general significantly different in different environments, facilitating localization. In Wi-Fi fingerprinting, the user’s location is determined by measuring the Wi-Fi RSS of the surrounding APs whose locations must not be known. In a first step, the off-line training phase, either signal propagation models for the specific environment are used or the RSS are measured at known reference locations to estimate and build up a fingerprinting database. In the second step, which is referred to as the on-line positioning phase, the current RSS measurements are used and matched with the RSS values in the database to estimate the users’ location. In the following, these two phases are discussed in more detail.

#### 3.1.1. Training Phase

In this first phase, a radio map database is created to characterize the properties of the RSS to every AP. This technique is usually more robust to environmental effects on the RSS than using RSS-based lateration (Section 3.2.1). This is because the fingerprinting approach constructs a search space according to either a simulated environmental propagation model, e.g., a model of the building, or directly measured RSS distributions in the area of interest. Using a simulated model, the characteristics of every object in the building have to be defined due to the fact that the Wi-Fi signal propagation is influenced by many factors (see Section 2.2.1). Physical models, such as ray tracing, according to the given environmental conditions, simulate the distribution of the RSS values.

A very simple empiric model is the so-called one-slope model, which is based on the principle on the Free Space Path Loss (*FSPL*) (see Equation (2)) of the signals [15]. The damping depends thereby only on the logarithmic distance between the transmitter and receiver and the reference RSS in the form:

(4) $$P\left(d\right)=P_{0}+10\cdot \gamma \;*\;{log}_{10}\left(d\right)$$

where *P* is the received empirical RSS, *P*_0_ is the reference RSS in 1 m distance, γ is the damping factor, and *d* is the distance between the transmitter and receiver.

Typical examples for path loss coefficients using the one-slope model are summarized in Table 4. The damping factor γ can vary significantly as indicated, which can then cause large errors for RSS to range conversions. As experimentally demonstrated by Retscher and Tatschl [15], an improved solution can be achieved if the coefficients *P*_0_ and γ are estimated empirically using a least-squares adjustment from the RSS measurements.

For estimation of the damping of walls and modeling of the *FSPL*, special semi-empiric models, such as the multi-wall model [34,35], can be applied. Semi-empirical means in this regard that the current surroundings are incorporated in the signal propagation estimation model. The multi-wall model considers the damping characteristics of existing walls between the transmitter and receiver whereby the direct path between them is treated. The authors in [34] have introduced a Multi-Wall Multi-Floor (MWMF) propagation model to generate a discrete RSS radio map (virtual fingerprints) relying on RSS prediction. Their experiments in multiple independent testbeds showed that this model outperforms in terms of accuracy in comparison to simpler propagation models. Moreover, the adoption of this model allows a seven-fold reduction in the number of measurements to be collected in the system training phase while achieving the same accuracy of a traditional fingerprinting system.

Basically, the multi-wall model in its simplest form can be realized by:

(5) $$P{\left(d\right)}_{rec}=P_{0}+10\cdot \gamma \;*\;{log}_{10}\left(d\right)+\sum_{i=1}^{n}D_{i}$$

where $P{\left(d\right)}_{rec}$ is the received RSS and $D_{i}$ is the damping value of the *i*-th wall.

In general, more realistic results can be achieved compared to the one-slope model in simple environments. However, the limitations of this model are soon reached if small-scale structures, such as corridors, columns, narrow staircases, etc., and different materials in the walls exist, such as reinforcement of the concrete, etc. [15,21]. As seen in the third case study (Figure 17 in Section 4.3), in very complex environments, the multi-wall model did not outperform the one-slope model. The main reason for this negative result is that the multi-wall model considers only the damping characteristics of existing walls between the transmitter and receiver whereby the direct path is only modeled. Multipath, refraction, and diffraction are out of consideration, as they cannot be modeled easily in practice.

Higher positioning accuracies are usually achieved when previously measured RSS values are used to establish the fingerprinting database. The advantage of constructing the database in that sense is that it can be used to consider a great number of detrimental effects from the surrounding environment, such as reflections and obstructions, into the fingerprinting radio maps, which thus increases the accuracy for finding the best matching location based on RSS measurements in the positioning phase.

The construction of a fine radio map is essential to obtain an acceptable positioning accuracy. The creation of an empirically determined radio map starts with the classification of reference points on the basis of a building model. Care should be taken to ensure that these reference points are well distributed throughout the building. A fingerprint represented by an RSS scan $s_{RP_{i},t}$ which was measured on a reference point $RP_{i}$ at time instance t, contains hence the measured RSS values $rss_{AP_{j}}$ of N APs in the form:

(6) $$s_{RP_{i},t}=\left[\begin{array}{c} rss_{AP_{1}}\\ rss_{AP_{2}}\\ \vdots \\ rss_{AP_{N}} \end{array}\right].$$

**Figure 7:**
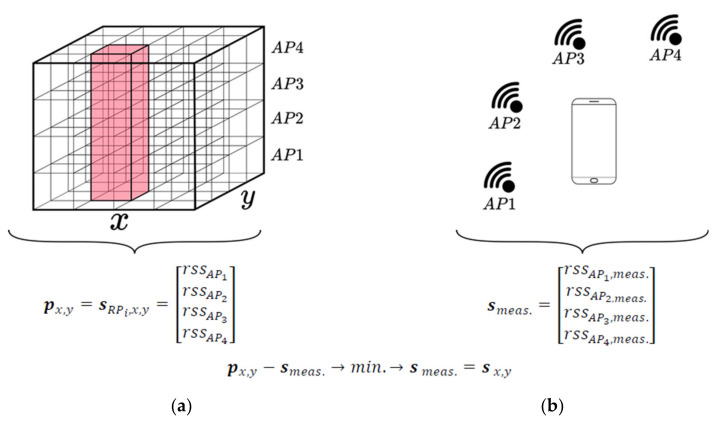
Positioning approach for the example of four visible APs with (**a**) measurements from the off-line training phase and (**b**) current measurements in the on-line positioning phase.

Then, the measured RSS are assigned to the corresponding APs in the database. If multiple scans are performed at a reference point, the database consists of all scans measured at each point. However, it can happen that the number of received signal strengths for each scan is different, e.g., because an AP is temporarily unavailable or the signal is too weak. This fact can lead to problems in localization, certainly in the case if RSS values of different APs occur in the currently observed fingerprint in the positioning phase and they are differing from the fingerprint in the radio map obtained from the training phase (compare Section 2.2.7). Figure 7 illustrates the positioning approach for the example that four APs are stored in the database represented by a datacube and four APs can be sensed in the on-line positioning phase with their current RSS measurements $s_{meas.}$. The chosen datacube is particularly a 3D array of radio maps of the sensed APs allowing the examination of the interrelations of the three quantities easily. Here, the datacube has two spatial axes and a vertical AP axis. It is created by stacking radio maps vertically onto each other. The spatial axes share the same properties with the pixel grid. This allows neatly extracting an RSS vector for any position by querying the spatial axes and retrieving all the values from the AP axis (Figure 7). Figure 9 shows an example how this can look if the radio maps are stacked in the datacube.

Interpolation algorithms have to be employed to generate a fine-grid RSS distribution for the radio map. Thereby, the following rule applies: the closer the grid reference points are, the higher the resolution of the radio map. For the radio map interpolation algorithms, such as the nearest neighbor (NN), Kriging and polynomial regression methods or even spline functions are commonly employed. The first approach follows a Delaunay algorithm based on Voronoï cells, allowing conducting the estimation of all fingerprints from the APs. Note that Delaunay and Voronoï concepts are linked because the Delaunay triangulation is the dual problem of the Voronoï diagram. The operational principle of the Voronoï/Delaunay method is that it uses the natural nearest neighbor (NN) interpolation [36], giving a smooth approximation of the interpolated function [37,38] as it considers also signal fading parameters. Therefore, it is superior to the implementation simplicity but not always as accurate as other algorithms. Kriging is an interpolation method based on the assumption that the interpolated values are spatially correlated and that they change continuously [39,40]. It is best used when the data have a strong trend and the trend can be modeled, such as large-scale RSS propagations in a Wi-Fi system. In the case of polynomial regression, the relationship of the variables is modeled using high-order polynomial functions [41]. Both Kriging and polynomial regression interpolations provide usually compatible results. The patterns of the detailed RSS variations are essential to conduct the matching in the positioning phase.

In the following, the principle of operation of the Voronoï interpolation approach is briefly reviewed. A Voronoï diagram consists of so-called Voronoï cells, which are defined by segments drawn between two points. Starting from a cloud of points, only some points are going to be linked between each other [37,38]. More precisely, the Voronoï/Delaunay method uses the nearest neighbor interpolation [36], giving a smooth approximation of the interpolated function. More accurate than linear interpolation, the building of the Voronoï tessellation brings an advantage in the following two steps: (1) to quickly estimate the localization of all the APs and (2) to estimate signal fading parameters. Thus, the Voronoï/Delaunay algorithm provides the possibility to interpolate the RSS function. In other words, it is going to perform a smooth interpolation of the function *f* (*x*,*y*) = RSS where (*x*,*y*) represents the position coordinates of a point *P* located in the radio map (in 2D) and RSS represents the value of the RSS in dBm for this point. Above all, a function is needed to interpolate. This function can form a base to estimate the RSS everywhere on the radio map. Thus, based on those APs, the function can be built for the interpolation. If the true distance between certain APs (at least two in the 2D case) is known, then the relationship between the RSS values that are received and transmitted to the range can be estimated (compare Equation (34) in Section 3.2.3), resulting in the range that the signals have traveled. This approach leads to the concept of Differential Wi-Fi developed by the author of this contribution. Further details can be found in Section 3.2.3 and in [15]. Experimental results are presented in the third case study in Section 4.3.

**Figure 8:**
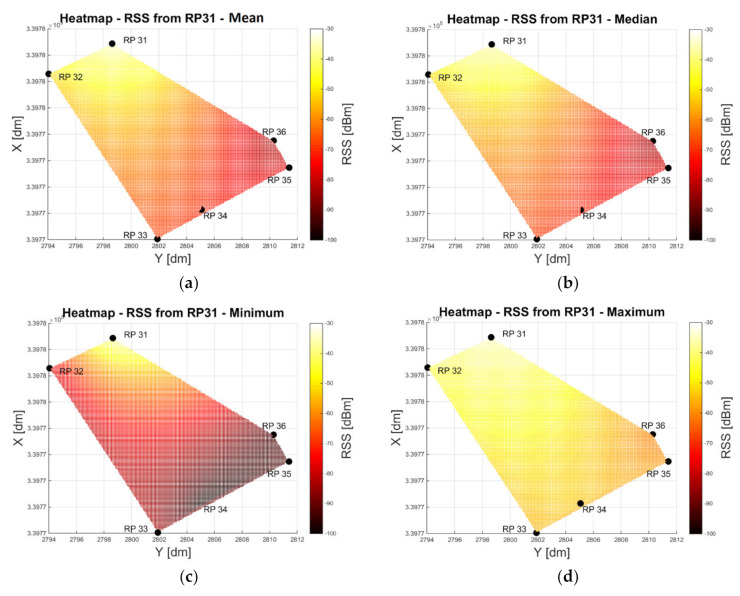
Radio maps of RSS distributions in the entrance area of a multi-story office building with (**a**) arithmetic mean, (**b**) median, (**c**) minimum, and (**d**) maximum RSS of four orientations. For the location of the six Raspberry Pi units (RPs) and the test locations, see Figure 14a.

Figure 8 shows the median, arithmetic mean, minimum, and maximum RSS value radio maps (or heat maps) in an entrance area of a multi-story office building. Six Raspberry Pi units (RPs) serving as APs were distributed in the area surrounding the test locations (see Figure 14a for their location). The spatial conditions are clearly recognizable; the signals of RP 31 can propagate more toward RP 34 than toward RP 35. Moreover, the signals to RP 35 decrease much more than to RP 34, with large differences between the minimum and maximum RSS values. The reason for this can again be attributed to the high fluctuations of the RSS at the beginning of the measurements (compare Figure 4).

**Figure 9:**
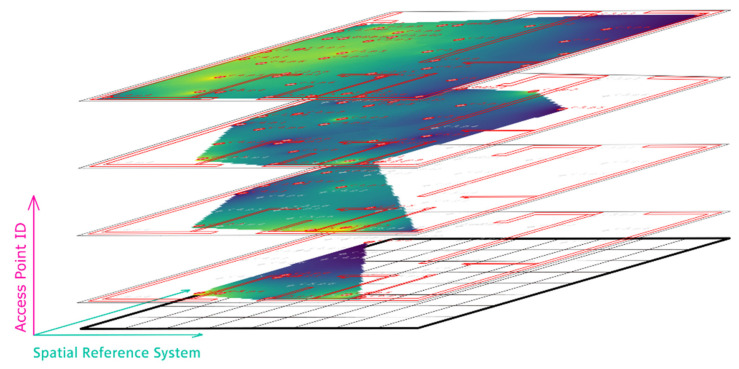
Example of a radio maps stack in the datacube for four sensed APs.

For the radio maps stacked in Figure 9, moving least square (MLS) interpolation was performed. This interpolation method moves from one interpolation point to another fitting a simplex through neighboring sample points. A simplex is a surface that is easily describable by few parameters, typically a plane or hyperbolic paraboloid. The validity of this local model is limited solely to that interpolation point, i.e., the certain pixel. While fitting, one can fine-tune the results of the method by introducing a weighting function, which determines the amount of influence that each sample point exerts on the fit. Afterwards, the method moves to the next interpolation point and executes the same procedure again. The great advantage of this method is that it is very general and therefore highly customizable. Three parameters can be adjusted leading to substantially different results, i.e., the form of the simplex, the neighborhood, and the weighting model. In the case shown in Figure 9, natural K-nearest neighbor (KNN) (compare Section 3.1.3) or K-quadrant neighbors interpolation based on the Delaunay triangulation was applied. The K-quadrant neighbors are used, which are represented by the selection of the KNN in each of the quadrants to prevent one-sided interpolation. This approach can be further modified by dividing the quadrants into octants by bisecting the four quadrants diagonally. The advantage of this method is that the whole set of sample points is relatively effectively considered whilst deciding which points are relevant for the fitting.

However, the system training, can be a time-consuming task with high effort for building up the RSS database, especially if RSS observations are carried out in a regular grid inside a building for the interpolation of a fine-grid radio map. An approach introduced by Hofer and Retscher [42] aimed at time reduction for system training by measuring RSS values only along defined user trajectories outdoors or inside the building and not in a regular grid. Trajectories are defined with waypoints at decision points, such as crossings of corridors, and in regular distances. User orientations are thereby measured only in the possible directions of movement. Then, the workload can be reduced by a factor of 4. In a further study, Retscher and Leb [29] derived the radio maps from kinematic system training measurements, i.e., these were performed while walking along specific trajectories in the area of interest. Then, these radio maps were interpolated using the aforementioned approaches. The resulting accuracies were similar to static training measurements at waypoints while saving a lot of time. To apply a crowdsourced approach for radio map creation is a further logical solution.

#### 3.1.2. Positioning Phase

The location of the user is determined in the on-line positioning phase with current RSS measurements and their match to the fingerprinting database (or radio map). Several positioning algorithms based on pattern recognition exist [2], which are either deterministic or probabilistic approaches. Deterministic location estimation is based on the similarity of the RSS measurements and the fingerprints in the database. Each RSS sample is not used separately, but the sample averages of different transmitters are collected into a vector and used to estimate the mobile device’s location [43]. In general, the probabilistic approach [44] exploits the sample of measurements collected during the training phase more efficiently than the deterministic methods. The idea is to compute the conditional probability density function (PDF) of the unknown position. In the following, the operational principle and the commonly used approaches are discussed.

#### 3.1.3. Deterministic Fingerprinting Approaches

The simplest ways to determine the location are the nearest neighbor (NN), *K*-nearest neighbor (KNN), or the weighted *K*-nearest neighbor (WKNN) algorithm where *K* reference points in the radio map are compared to the observed measurements to select *K* reference points with the nearest RSS values. Vector distances, most commonly based on the Euclidean norm, are used as a distance measure [45,46,47]. The vector distance d between the observed fingerprint $f_{obs}$ and the respective fingerprint in the radio map $f_{map}^{i}$ is calculated, and then, the position with the shortest distance in the radio map, i.e., the nearest neighbor, yields the unknown location:

(7) $$X_{NN}=argmin\;d\left(f_{map}^{i}\;,f_{obs}\right).$$

Then, the Euclidean vector distance results in the form:

(8) $$d\left(f_{map}^{i},\;f_{obs}\right)=\sqrt{\sum_{j=1}^{n}{\left(f_{obs}^{j}-f_{map}^{i,j}\right)}^{2}}.$$

Wi-Fi scans are recorded using their unique MAC address and the associated reference point (RP) location coordinates in a fingerprinting database (DB) in the training phase. At the end of this phase, every RP position i is described by a RSS vector $RSS_{i}$ which consists of the RSS values $Si_{APx}$ of the visible APs AP1 to APn (see Equation (9)). In the training phase, any desired number of RPs and scans can be measured and stored. The nearest neighbor (NN) algorithm is a deterministic approach to obtain the location of a user in the positioning phase [48] by comparing current RSS measurements with the RSS values in the DB. For positioning, Wi-Fi RSS scans can be carried out at any place. From it, the vector $RSS_{m}$ (see Equation (10)) is provided, and the Euclidean distance $d_{m,i}$ to all vectors $RSS_{i}$ in the DB is calculated (Equation (11)). Then, the position is chosen that has the smallest distance from the values in the DB. In other words, the certain position is chosen that represents the nearest neighbor to the vector $RSSI_{m}$.

(9) $$RSS_{i}=\left[Si_{AP1},\;Si_{AP2},\ldots ,Si_{APn}\right]\;\ldots \;\mathrm{RSS}\;\mathrm{vector}\;\mathrm{for}\;\mathrm{training}\;\mathrm{phase}$$

(10) $$RSS_{m}=\left[Sm_{AP1},\;Sm_{AP2},\ldots ,Sm_{APn}\right]\ldots \;\mathrm{RSS}\;\mathrm{vector}\;\mathrm{for}\;\mathrm{positioning}\;\mathrm{phase}$$

(11) $$d_{m,i}=\sqrt{{\left(Sm_{AP1}-Si_{AP1}\right)}^{2}+{\left(Sm_{AP2}-Si_{AP2}\right)}^{2}+\cdots +{\left(Sm_{APn}-Si_{APn}\right)}^{2}}$$

**Figure 10:**
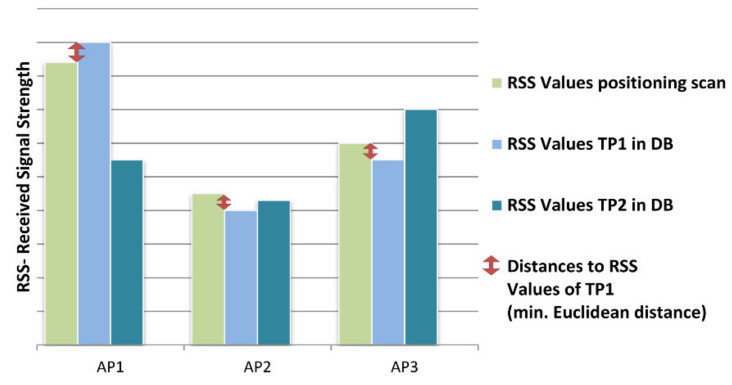
Allocation of positioning scans to the stored RSS scans in the training fingerprinting database DB [42].

This calculation has to be done for all possible locations. Figure 10 illustrates the principle idea for the definition of the distance relationship between the DB of the training phase and the positioning phase. In the simplified case shown in the figure, the RSS values for two test locations TP 1 and TP 2 are stored in the DB where the allocation of the positioning scans with the DB is specified regarding their corresponding minimum distance. If the Euclidean distance as a matching strategy is used, then the scan is allocated to TP 1 in this simple example [42].

In the positioning process, a weighted Euclidean distance value $eukdis_{Weig,i:PosID}$ for an RSS vector i is calculated in the following form:

(12) $$\bar{weighted}=\left[w_{AP_{1}},\ldots ,w_{AP_{n}}\right]$$

(13) $$eukdis_{Weig,i:PosID}=\sqrt{{\left(Sm_{AP1}-S_{i:PosID,\;AP_{1}}\right)}^{2}\cdot w_{AP_{1}}+\cdots +{\left(Sm_{APn}-S_{i:PosID,\;AP_{n}}\right)}^{2}\cdot w_{AP_{n}}}.$$

The success of the fingerprinting-matching approach using the NN algorithm in the positioning phase can be described by using a so-called matching rate. It is defined as given in Equation (14).

(14) $$\mathrm{matching}\;\mathrm{rate}\;\mathrm{MR}\;=\frac{\mathrm{number}\;\mathrm{of}\;\mathrm{correctly}\;\mathrm{assigned}\;\mathrm{RSS}\;\mathrm{scans}\;\mathrm{to}\;\mathrm{RPs}}{\mathrm{total}\;\mathrm{number}\;\mathrm{of}\;\mathrm{all}\;\mathrm{RSS}\;\mathrm{scans}\;\mathrm{in}\;\mathrm{positioning}\;\mathrm{phase}\;}.$$

Equations (9)–(14) describe the main relationship for the fingerprinting process using the NN based on the Euclidean vector distance. If several positions with a small distance are found in the NN algorithm, a set of points with the *K*-smallest distances can also be selected. The value K≥2 can be an arbitrarily selected number or can be determined by a threshold value for a certain minimum distance. In this KNN approach, the location of the user is usually the center of gravity of the *K* positions $X_{NN,j}$ with the k-smallest vector distances:

(15) $$X_{KNN}=\;\frac{1}{K}\sum_{j}^{K}X_{NN,j}.$$

**Figure 11:**
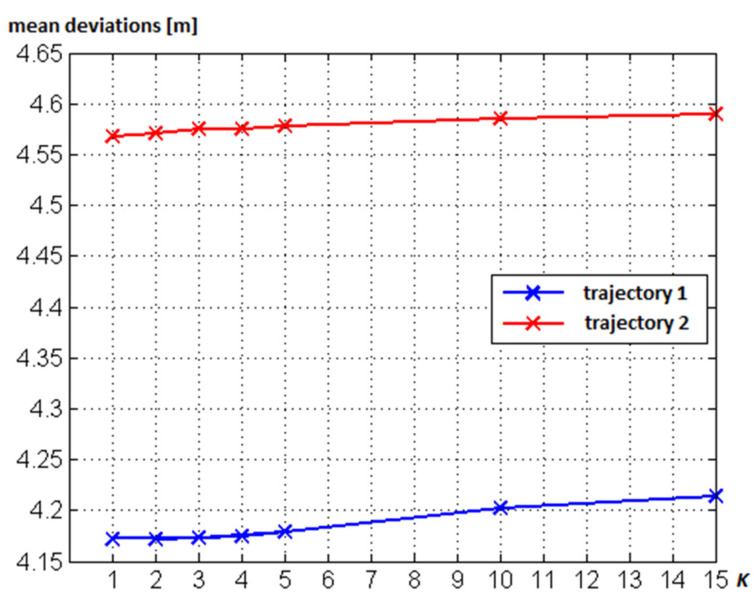
Resulting mean deviations from the ground truth of the *K*-nearest neighbor (KNN) approach for different *K* values ranging from 1 to 15. The measurements were carried out along two different trajectories located on the ground floor of a multi-story office building (see Figure 15 for their location).

The KNN method can still be optimized by calculating a weighting for each *K* fingerprint, on the basis of which the center of gravity of all *K* fingerprints can be estimated as the location of the smartphone user, i.e., the WKNN approach. Huang [49] stated that the positioning accuracy increases if a *K* value of up to 10 in maximum is used, and then it decreases again. An empirical analysis of different *K* values by Retscher and Leb [29] along two trajectories in the test area depicted in Figure 15 revealed that no significant improvement of the mean deviations from the ground truth are achieved by increasing *K* while measuring along waypoints of predefined trajectories. Figure 11 shows the results of the KNN approach with different *K* values ranging from 1 to 15. Contrary to the expectations, the mean deviations of the resulting position fixes from the ground truth even increased slightly as the value of *K* increases over a value of 5. However, this increase was only in the centimeter range.

As a suitable alternative, also machine learning algorithms, such as neural networks, random forest, decision trees, or support vector machines (SVM), may be applied in deterministic fingerprinting [50]. In Figuera et al. [51], a good overview and discussion of neural networks and SVM algorithms for Wi-Fi positioning can be found. However, the authors claim that commonly employed learning algorithms do not significantly outperform the KNN approach in most cases. They achieved an improvement of the performance of the employed SVM algorithm only if a priori information within the learning machine, such as using the spectral information of the training data set, and a complex output taking into account the advantage of the cross information in the two dimensions of the location, are used in addition. A higher performance improvement for fingerprinting is mainly only achievable if probabilistic approaches for positioning are employed. Two selected approaches that were employed in our studies are described in the following section.

#### 3.1.4. Probabilistic Fingerprinting Algorithms

Bayesian filtering employing probabilistic techniques is one strategy to estimate the states of a dynamic system with measurement noise. It is also applicable to sensor integration consisting of many different types of measurements to achieve higher accuracy. While using a conditional PDF of the unknown position, usually, a uniform prior distribution is considered. Using Bayes’ theorem (see e.g., [52]) and the measurements, the posterior PDF can be calculated because of the fact that the fingerprints contain information about the signal characteristics. Several approaches for the calculation are available, e.g., the histogram method and the kernel method (see [44,45]). For instance, a particle filter (see [53]) can be used for the estimation of the most likely location of the user. In the following, two approaches are briefly discussed.

As conditional probabilities are applied, the location with the highest probability then results in the searched position in the form:

(16) $$X=\arg\;\max\;p\left(f_{map}^{i}\;|\;f_{obs}\right)$$

where *X* is the conditional probability that the user has received the current on-line measurement $f_{obs}$ at the position $f_{map}^{i}$. Thereby, the probability is based on Bayes’ theorem:

(17) $$p\left(f_{map}^{i}\;|f_{obs}\right)=\frac{p\left(f_{obs}|f_{map}^{i}\;\right)\;p\left(f_{map}^{i}\right)}{p\left(f_{obs}\right)}=\frac{p\left(f_{obs}|f_{map}^{i}\;\right)\;p\left(f_{map}^{i}\right)}{\sum_{j}^{N}p\left(f_{obs}|f_{map}^{i}\;\right)\;p\left(f_{map}^{i}\right)}.$$

The probability density function $p\left(f_{map}^{i}\right)$ is the a priori probability of the user’s location over the entire area, and it is usually assumed to be normally distributed. With this value, it is possible that positions where users are more frequent are assigned a higher probability. Usually, $p\left(f_{map}^{i}\;\right)=\;\frac{1}{N}$ is assumed, because there is no knowledge about the user position in advance and all points N in the radio map can be assumed with the same probability. Therefore, it is sufficient to calculate $p\left(f_{obs}|f_{map}^{i}\;\right)$, since the remainder of Equation (17) is thus constant. The goal now is to maximize $p\left(f_{obs}|f_{map}^{i}\;\right)$, i.e., to find the position in the radio map where it is most likely to measure $f_{obs}$:

(18) $$X_{ML}=\arg\;\max\;p\left(f_{obs}|f_{map}^{i}\;\right).$$

Then, the fingerprint with the highest probability is assumed to be the position of the smartphone user. This method is referred to as maximum likelihood (ML) [54]. If we assume the normal distribution again, then $p\left(f_{obs}|f_{map}^{i}\;\right)$ can be calculated as follows (derivation in [55]):

(19) $$p\left(f_{obs}|f_{map}^{i}\;\right)=\frac{1}{{\left(2\pi \right)}^{\frac{N}{2}}{\left|C_{ff_{map,i}}\right|}^{\frac{1}{2}}}exp^{\left[-\;\frac{1}{2}\;{\left(f_{obs}\;-\;f_{map}^{i}\;\right)}^{T}C_{ff_{map,i}}^{-1}\left(f_{obs}\;-\;f_{map}^{i}\;\right)\right]}$$

where N is the number of received signal strengths for the fingerprint $f_{map}^{i}$ and $C_{ff_{map,i}}$ is its empirical covariance matrix. Since the goal is to maximize $p\left(f_{obs}|f_{map}^{i}\right)$, the exponent in Equation (19) must be minimized. This exponent is identical with the so-called Mahalanobis distance $d^{M}$ except for the constant factor −0.5:

(20) $$d^{M}\left(f_{map}^{i},\;f_{obs}\right)={\left(f_{obs}\;-\;f_{map}^{i}\right)}^{T}C_{ff_{map,i}}^{-1}\left(f_{obs}\;-\;f_{map}^{i}\right).$$

If the covariance matrix is the unit matrix, the Mahalanobis distance $d^{M}$ therefore corresponds to the Euclidean vector distance, which is most commonly used in the deterministic fingerprinting approach (see Equation (8)). As the inverse of the covariance matrix is the weight matrix, the weighted square sum of the RSS differences (between off-line training and on-line positioning phase), whereby the weights are inversely proportional to the variances of the corresponding fingerprints, is calculated for the Mahalanobis distance. A large number of studies have shown that probabilistic fingerprinting offers a higher accuracy than the deterministic approaches in indoor positioning, as they take better account of signal fluctuations. For instance, Leb and Retscher [56] could prove that in kinematic positioning where the pedestrian user walked with normal step speed, positioning accuracies of 2 m on average are achievable while using the Mahalanobis distance. This type of accuracy is comparable with commonly employed algorithms where a static or stop-and-go mode for localization is applied.

A further Bayesian filtering approach is to employ a Hidden Markov Model (HMM) framework [12]. Compared with other Bayesian filtering technologies, a HMM has the advantages of non-Gaussian assumption and computation efficiency [57]. The hidden states in the HMM framework can be Wi-Fi fingerprints [19] or the reference points [58], and with Pedestrian Dead Reckoning (PDR), the state transition matrix can also be generated. Then, the HMM is more feasible to estimate the motion with free moving restrictions [59]. The graph structure, which represents the environment defined by vertices and edges, is proposed in He et al. [59]. In the following, a brief summary of the HMM approach employed following the theory introduced by Rabiner [60] is given.

Let *s*_1_, *s*_2_, …, *s_t_* be the sequence of hidden states in the state set *S*, which constitutes the user moving trajectory that is specific in the case under consideration at time *t*. Given an observed Wi-Fi RSS sequence *O* = *o*_1_, *o*_2_, …, *o_t_* up to time *t*, the model is characterized by parameters $\Lambda =\left\{A,\;B,\pi \right\}$ where **A** is the transition probability matrix characterizing the state transition probability independent of time and **B** is the emission probability matrix characterizing the observation probability of an observation given its state [12]. They are defined by the following equations:

(21) $$A=\left[P\left(s_{t}|s_{t-1}\right)\right]$$

and

(22) $$B=\left[P\left(o_{t}|s_{t}\right)\right].$$

π is the initial state probability and it is normally set to:

(23) $$\;\;\;\;\;\pi =P\left(s_{1}\right)=\frac{1}{N}$$

where *N* is the number of states in *S*.

In the HMM-based tracking system, the Viterbi algorithm (see e.g., [61]) can be applied to calculate the maximum posteriori estimate of the path given the observation sequence.

The fingerprinting approach normally averages the RSS measurements for each AP in stored signatures, assuming a unimodal RSS distribution. Another drawback for fingerprinting is that it is not consistent to dynamic environment changes. Meanwhile, using a histogram of RSS for a signature at a reference point instead may offer a more accurate description of the environment, as a histogram represents various levels of RSS by storing a large number of signatures [62]. The histogram method is closely related to the discretization of continuous values to discrete ones. Firstly, select a room or a part of the corridor in a building and take many RSS measurements in this area. Then, quantize the measurements for each AP into *m* values, i.e., *m* bins, which is a set of non-overlapping intervals that cover the whole range of the RSS measurement. Thus, the quantized measurements, i.e., RSS levels for each AP, are received. For simplicity, equal-width bins are used in the following. Now, the probability of each bin given the user is in the room is the normalized count of measurements in that bin from inside the room as given in:

(24) $$\;\;\;\;\;P\left(rss_{1}\in ith\;\mathrm{bin}\right)=\frac{\mathrm{count}\;\left(ith\;\mathrm{bin}\right)}{\mathrm{size}\;\mathrm{of}\;\mathrm{training}\;\mathrm{data}}.$$

In the training phase, a Wi-Fi fingerprint for each room or section of a building, referred to as cell, is created following this relationship. In this way, the labor-consuming workload can be reduced relatively since the grid step can be skipped, as it is not necessary to measure or scale the area nor know the true dimension of the floor. However, multiple scans are still needed at multiple reference points to create the histogram for each AP. In this stage, RPs within the room are chosen randomly, and Wi-Fi signals at each point are collected regardless of the user direction [60]. Then, the online navigation phase matches current RSS measurements to the room fingerprints to determine the user’s most likely location. It uses maximum likelihood classification for this matching process. The likelihood of a vector of measurements $Z=\left\{z_{1},z_{1},\ldots ,z_{n}\right\}$ with $n\in N_{ap}$ for a certain room within the building $l_{k}$ with $k\in N_{cell}$ that the user is in, where it is assumed that the sensed RSS from the APs are independent, is described according to the Bayes’ rule by:

(25) $$\;\;\;\;\;p\left(l_{k}|Z\right)=\frac{p\left(Z|l_{k}\right)\;p\left(l_{k}\right)}{P\left(Z\right)}$$

and

(26) $$\underset{k}{\max}\left(p\left(l_{k}|Z\right)\right)=\underset{k}{\max}\left(p\left(Z|l_{k}\right)\right)=\underset{k,\;i\in m}{\max}\left(\prod_{n=1}^{N_{ap}}\mathrm{hist}(Z_{n}\in ith\;\mathrm{bin}\right).$$

Using the histogram matrix computed at the training phase as a look-up table, then the emission (observation) probability for each timestamp is computed. In this way, a room-based histogram probabilistic method is realized [12,63]. If one considers that within one Wi-Fi RSS scan, some APs are non-visible, a value of ZERO is assigned to them, and they are not included into the emission probability computation. For example, if a Wi-Fi scan at some timestamp is $O=\left(O_{1},O_{2},\;O_{3},\;O_{4},O_{5},O_{6},O_{7}\right)$ where $O_{4}$, $O_{5}$ and $O_{6}$ are ZERO means that AP 4, AP6, and AP7 are non-visible, then the probability for the measurement at *RPj* is computed as follows [63]:

1. The observation is a vector of length *m* at each test point where m≤7 and $O=\left(O_{1},O_{2},\;\ldots ,\;O_{m}\right)$;
2. *m* is a visible AP; in the case of a non-visible AP *RSS* = 0 dB is assigned;
3. Equation (27) yields the emission probability for a given observation:
  (27) $$P\left(O|RP_{j}\right)=\prod_{i=1}^{m}P\left(O_{i}|RP_{j}\right)$$
  with j∈1:10.

For example given the example above, the emission probability results in:

(28) $$P\left(O|RP_{j}\right)=Hist_{AP1}\times Hist_{AP2}\times Hist_{AP3}\times Hist_{AP5}.$$

As an alternative to histograms, the Weibull function may be applied [1]. The pattern recognition and different Bayesian approached require the knowledge of the spatial dependency between the positions and the RSS observables, which need to be trained before applying it for positioning. As aforementioned, a high workload is required if a large number of training measurements for generating the fingerprint database and a fine-grid radio map have to be established. In order to obtain a better approximation of the PDF by using a small number of training measurements, Liu et al. [64] used a Weibull distribution function to represent the probability of the RSS measurements. For example, it has been widely used to model the RSS of radio propagation in radar systems and wireless communications [65]. The Weibull distribution function $f\left(x;\lambda ,\;k,\;\theta \right)$ is given as follows:

(29) $$f\left(x;\lambda ,\;k,\;\theta \right)=\left\{\begin{array}{c} \frac{k}{\lambda }{\left(\frac{x-\theta }{\lambda }\right)}^{k-1}\;*\;\exp\left(-{\left(\frac{x-\theta }{\lambda }\right)}^{k}\right)\mathrm{for}\;x\geq \theta \\ 0\;\;\;\;\;\;\;\;\;\;\;\;\;\;\;\;\;\;\;\;\;\;\;\;\;\;\;\;\mathrm{for}\;x<\theta \end{array}\right.\;$$

where λ is the scale parameter, *k* is the shape parameter, and θ is the shift parameter. *x* is the variable of the function. Then, the cumulate distribution function is defined as:

(30) $$F\left(x\right)=1-\exp\left(-{\left(\frac{x-\theta }{\lambda }\right)}^{k}\right)\;.$$

For the Weibull function distribution, common statistics, such as the mean, the standard deviation, and the median can be used. Chen et al. [1] analyzed results of the difference between the Weibull function-based and histogram-based RSS probability densities. They concluded that for the used 19 RSS training measurements, the Weibull function results in a good approximation of the PDF. Liu et al. [64] demonstrated that the Weibull function approach can increase the positioning accuracies compared to the histogram-based approach by 20%.

### 3.2. Lateration

As a conventional algorithm employed is surveying, lateration can be employed in Wi-Fi positioning either based on RSS or most recently on Round Trip Time (RTT) measurements in the new Wi-Fi standards. If three transmitters, i.e., APs, are involved in the measurements, it is referred to as trilateration, and if there are more than three, it is referred to as multi-lateration, respectively. In this section, first, the RSS-based techniques are described followed by a discussion of the concept of Wi-Fi RTT lateration and a differential approach that can be applied for both techniques.

#### 3.2.1. RSS-Based Lateration

In RSS-based techniques, lateration is based on the signal propagation of the RSS, which varies with changes of the range to the AP. Theoretically, the RSS decreases with the transmitted energy propagating into space. A number of models, termed path loss models, have been developed to establish the RSS to range relationship. Their principle is that the trend can be mathematically modeled. One of the simplest models that describes the decreasing trend without the effects from reflections and obstructions is presented as the log-distance path loss model *PL*(*d*) as given in:

(31) $$PL\left(d\right)=PL\left(d_{0}\right)+10\;*\;\gamma \;*\;\log\left(\frac{d_{0}}{d}\right)\;\mathrm{with}\;d\geq d_{0}\geq d_{f}$$

where *d* is the distance between the transmitter and receiver, *PL*(*d*) the path loss at *d*, $d_{0}$ is the reference distance, $d_{f}$ is the Fraunhofer distance, and γ is the path loss exponent. Thereby, the Fraunhofer distance defines the boundary of the region.

**Figure 12:**
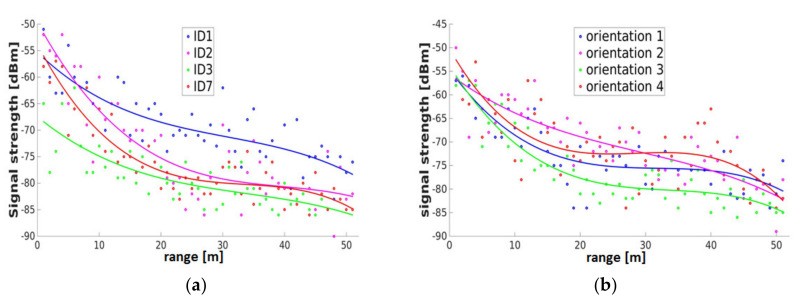
RSS to range relationship of the mean of all four orientations (**a**) for four smartphones (ID1, ID2, ID3, and ID7) and (**b**) in all four orientations for smartphones ID7 on the right.

Some typical examples of the path loss exponent γ can be found in the literature (see e.g., [66]). Usually, γ equals 2 in free space, which aligns the log-distance path loss model well with the free space propagation model (compare Equation (2)). In other environments and non line-of-sight (NLoS) conditions, γ can have values between 2 and 6. However, in the indoor line-of-sight (LoS) areas, γ can have values less than 2. This is caused by the surrounding structures forcing the radio-frequency signals to propagate along the directions of the structures instead of propagating uniformly into the space. Retscher et al. [67] analyzed the path loss in an open area and along a corridor. As seen by the authors in the open area, the path loss pattern is close to the log-distance pattern. However, along a corridor in a building, they saw a linear pattern. Figure 12 shows examples of the relationship between the RSS and the range between the transmitter and receiver for a mobile client using a smartphone. The ranges between the transmitter and receiver can be calculated according to the RSS by inverting the path loss model.

However, the limitations are that most of these theoretical models are subject to free space propagation or signal propagation in a simple environment with a limited number of reflectors and obstacles. Due to the fact that the environment can be tremendously complex, it is very difficult to deal with in the real world through the assumptions or conditions of the theoretical models. Thus, an important requirement is that the users’ devices must be calibrated to determine this relationship empirically. This can be done on calibration baselines. Figure 12a shows an example of observations of RSS values from four smartphones on a 50 m long calibration baseline with a spacing of 1 m between two APs. The measurements were performed in four orientations aligned to the axes of the building, which is a common practice in Wi-Fi positioning to encounter for the shielding of the signals due to smartphone users (see Section 2.2.4, Figure 3). A polynomial approximation of third order was fitted to the measured data. It can be observed in Figure 12 that the RSS are quite scattered around the approximated polynomial function. For ranges of up to 25 m, only a valid conversion of the RSS to a range can be made, as the gradient of the curve provides a sufficiently good relationship. Beyond this distance range, the polynomial functions are leveling off, and a definite conversion is not evident. If one looks at Figure 12b showing all four orientations of one smartphone, orientation 3 shows the lowest RSS values, which is as expected, because this is the orientation where the AP is located behind the user carrying out the survey. It is evident that the range to the RSS can be increased if an average of the four orientations is used. After conversion of the RSS into the ranges, the users’ location can be estimated using lateration. Due to the lack of consideration of short-term signal fluctuations in the path loss models, positioning accuracies are usually slightly lower than in location fingerprinting. They result mostly in the range of a few meters only [15].

#### 3.2.2. RTT-Based Lateration

With the latest generation of Wi-Fi hardware, the two-way Time-of-Flight between the APs and the mobile client can be measured. It is referred to as Round Trip Time (RTT). One advantage of this method is that the mobile device is both a transmitter and receiver at the same time, which means that there is no need for exact time synchronization between the smartphone and the AP. However, the exact time delay caused by the responder must be known, which is difficult to determine. This problem was solved with introducing the IEEE 802.11mc standard, which makes it possible to determine the turnaround time with sufficient precision [13,14,68,69]. The operational principle is as follows: the smartphone scans the APs in the surroundings and recognizes which of them are RTT-capable. Then, a request is made to the APs, and the AP responds with a so-called ping-pong protocol. First, a so-called FTM (Fine Timing Measurement) protocol is sent to the smartphone (ping). Then, the smartphone sends an acknowledgement back (pong). The transmitted and receiving time on each device is added to the protocol. In order for the smartphone to calculate the complete turnaround time, it needs four time stamps. For this reason, the AP sends out a package containing all four time stamps. Then, the smartphone calculates the travel time by subtracting the time stamps of the AP and the smartphone. Then, the difference between these two time stamps is the travel time it took to send the package from the AP to the smartphone and back again. Then, the travel time is multiplied by the propagation speed and divided by two to obtain the range between the AP and mobile device. Equation (32) describes the relationship if four measurements are carried out and the mean is calculated:

(32) $$t_{RTT}=\frac{1}{N}\left(\sum_{i=1}^{N}t_{4_{i}}-\sum_{i=1}^{N}t_{1_{i}}\right)-\frac{1}{N}\left(\sum_{i=1}^{N}t_{3_{i}}-\sum_{i=1}^{N}t_{2_{i}}\right)$$

where $t_{1_{i}}$ is the timestamp when the FTM framework is first sent by a Wi-Fi AP, $t_{2_{i}}$ is the timestamp when the FTM signal arrives at the smartphone, $t_{3_{i}}$ is the timestamp when the smartphone returns the acknowledgment (ACK) signal to the AP, $t_{4_{i}}$ is the timestamp when the ACK signal is finally received by the AP, *N* is the successful burst number (where *N* > 0, *N* < *B*) and *B* is the total burst number (i.e., burst size, *B* = 8 by selected default).

Generally, the protocol excludes the processing time on the smartphone terminal by subtracting $t_{3_{i}}-t_{2_{i}}$ from the total Round Trip Time $t_{4_{i}}-t_{1_{i}}$, which represents the time from the instant the FTM message is sent $t_{1_{i}}$ to the instant that the ACK is received ($t_{4_{i}}$). This calculation is repeated for each FTM-ACK exchange, and the final RTT is the average over the successful number of FTM-ACK bursts. The estimated range $D_{est}$ can be obtained through Equation (33):

(33) $$D_{est}=\frac{1}{2}\;*\;t_{RTT}+c.$$

If the ranges to at least 3 APs are measured, then the location of the user can be determined by means of lateration. Therefore, the prerequisite is that both the APs and the smartphone support the IEEE 802.11mc standard [13]. For Wi-Fi RTT, the smartphone does not need to connect to the APs, and only the smartphone is used to determine the range so that the privacy of the smartphone user is guaranteed [70]. Therefore, Wi-Fi RTT is a promising method enabling to determine the ranges even on the sub-meter level, and for the position, the achievable accuracy lies in the meter range [14]. However, this method is currently only available with a few smartphones on the market with Android version 9 or higher. For the planned implementation at TU Wien, hardware for the new APs will be based on low-cost computers, i.e., Raspberry Pi units (see the following Section 3.2.3). RSS-based approaches will still be needed since the coverage with new hardware cannot always be guaranteed. Therefore, a combination and integration of technologies as a hybrid solution will be appropriate.

#### 3.2.3. Differential Wi-Fi (DWi-Fi)

The basis of this technique is differential GPS (Global Positioning System), short DGPS, where reference stations (RS) whose coordinates are known are used to estimate range correction parameters for the ranges measured to GPS satellites or coordinate corrections derived from GPS range measurements for the RS. As in DGPS, reference stations are now deployed transmitting and scanning Wi-Fi signals. Introduced by the author of this contribution, this method was termed in analogy Differential Wi-Fi (DWi-Fi). The approach aims at a significant reduction of the effects caused by signal fluctuations on the positioning result. The principle of operation in more detail is as follows: The user applies the corrections derived from the continuous observations of the RSs in real-time to improve his current localization accuracy. In an RS network, so-called FKPs (Flächenkorrekturparameters) are estimated, representing a spatial and temporal model of the range corrections. Thus, the influence of Wi-Fi signal fluctuations (compare Section 2.2.1) can be reduced if the corrections are applied in real time at the user side in the case of the RSS-based approach. The RS were realized by low-cost computers, i.e., Raspberry Pi units, which serve at the same time as AP and RS.

**Figure 13:**
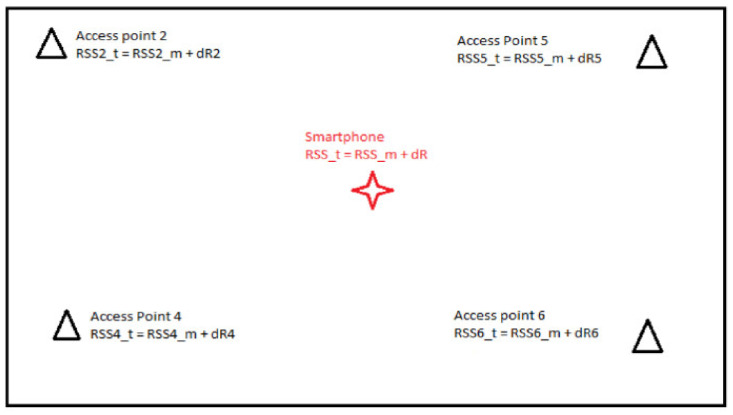
Differential Wi-Fi (DWi-Fi) operational principle with four APs.

Figure 13 illustrates the concept for four APs: 1, 4, 5 and 6. Then, the following Equations (34) and (35) are used for the calculation in this selected case:

(34) $$P_{received}=P_{transmitted}++20\log\left(\frac{\lambda }{4\pi d}\right)\;$$

(35) $$dR=w_{2}\times dR_{2}+w_{4}\times dR_{4}+w_{5}\times dR_{5}+w_{6}\times dR_{62}\;$$

where w are the weights for the different APs.

As reported in Retscher and Tatschl [15], higher positioning accuracies can be achieved than for commonly employed RSS-based lateration approaches; they were similar, as in fingerprinting. DWi-Fi is applicable for both RSS- and RTT-based lateration. If RTTs to APs are measureable, a further improvement with the differential approach can be expected.

### 3.3. Discussion and Assessment of the Selected Approaches

In the sections above, selected approaches and algorithms that are most commonly employed were presented. Apart from them, other possibilities exist having different advantages and disadvantages. Especially, the derivation and calculation of the Mahalanobis vector distance in the probabilistic approach is a promising and highly suitable solution. Thereby, nearest neighbor (NN), *K*-nearest neighbor (KNN), and *K*-weighted nearest neighbor (KWNN) approaches for matching of the fingerprints from the off-line training to the on-line positioning phase are employed. In general, in RSS-based techniques probabilistic fingerprinting outperforms the other methods. Thereby, the most promising are the Bayesian approaches. RSS-based lateration suffers from inherent difficulties in the RSS to range relationship and its conversion. Several models are commonly applied, mostly logarithmic path loss models. An improvement is achieved if reference stations (RSs) scanning and broadcasting Wi-Fi signals are deployed in the area of interest. This leads to the concept of DWi-Fi introduced by the author of this contribution.

As a novel approach that benefits from all advantages of the different techniques, the fusion of location fingerprinting and lateration was proposed by the author [71] to combine the advantages of both methods. As in the RSS-based DWi-Fi technique, RSS observations are continuously carried out at the RSs; they can be used to derive dynamically changing and updated radio maps in real-time to encounter for large temporal and spatial variations of the radio channel. From the radio maps arrays, e.g., stacked in a datacube (see Figure 9), correction parameters are derived to estimate the ranges between the RSs (serving at APs at the same time) and the user. Then, the location of the user is calculated via lateration. An assessment of the benefits and results yielded a significant performance improvement.

## 4. Kinematic Positioning Case Studies

The following four selected case studies highlight the achievable results of RSS-based Wi-Fi positioning. Mainly kinematic user localization along predefined trajectories is considered in the examples, as it is more challenging than in static and stop-and-go mode. Then, deviations from the ground truth on waypoints or the whole trajectory are analyzed. Fingerprinting and lateration approaches are compared.

### 4.1. Case Study 1: Combined Outdoor/Indoor Trajectory

**Figure 14:**
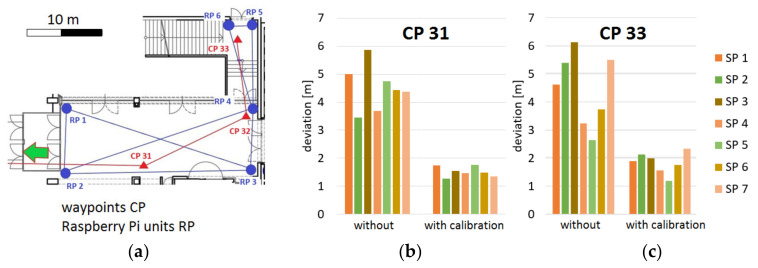
Indoor trajectory (**a**) and deviations from the ground truth without and with calibration for seven different smartphones (SPs) on two waypoints CP 31 (**b**) and CP 32 (**c**)

In the first case study, a combined outdoor/indoor trajectory leading to a multi-story office building was investigated. Figure 14a shows the entrance section of the building with different waypoints (referred to as checkpoints, or CP) and the location of six Raspberry units (referred to as RP) serving as APs and reference stations. Figure 14b,c show a comparison of the deviations from the ground truth on the trajectory waypoints CP 31 and 33 for seven different smartphones. Thereby, the user set a marker in the RSS recordings at each CP. As can be seen, the deviations can be significantly reduced if a calibration model based on a multivariate regression (see Section 2.2.5) is applied. In addition, the reduction of the influence caused by the heterogeneity of the smartphones is clearly visible.

### 4.2. Case Study 2: Continuous Indoor Trajectory Estimation

**Figure 15:**
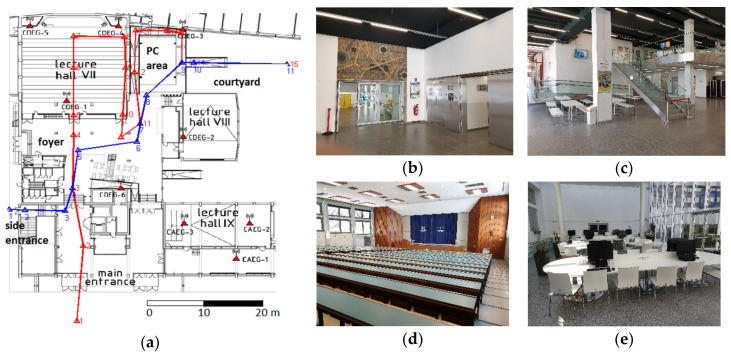
Two trajectories (**a**) in the tests site on the ground floor of a multi-story office building leading from outdoors through the main entrance (**b**) to the foyer (**c**), the classroom VII (**d**), and an area with desktop computers (**e**) to the inner courtyard.

Apart from static observations or in stop-and-go mode, more challenging continuous kinematic measurements along walked trajectories are analyzed in the following. Figure 15 shows two sample trajectories leading into the same office building either from the main or the side entrance through a foyer, classroom VII, and an area with desktop computers (PC area) to the inner courtyard of the building.

**Figure 16:**
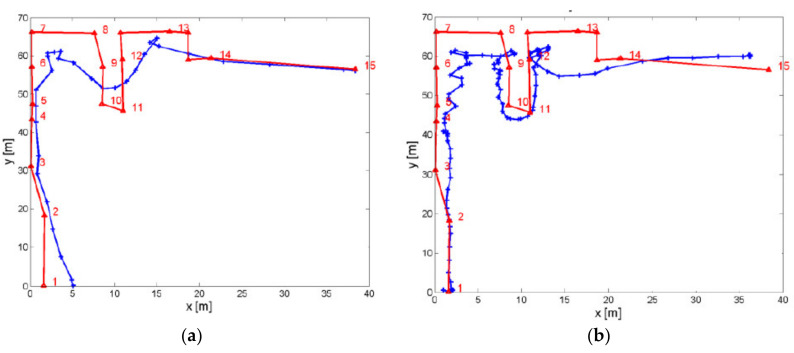

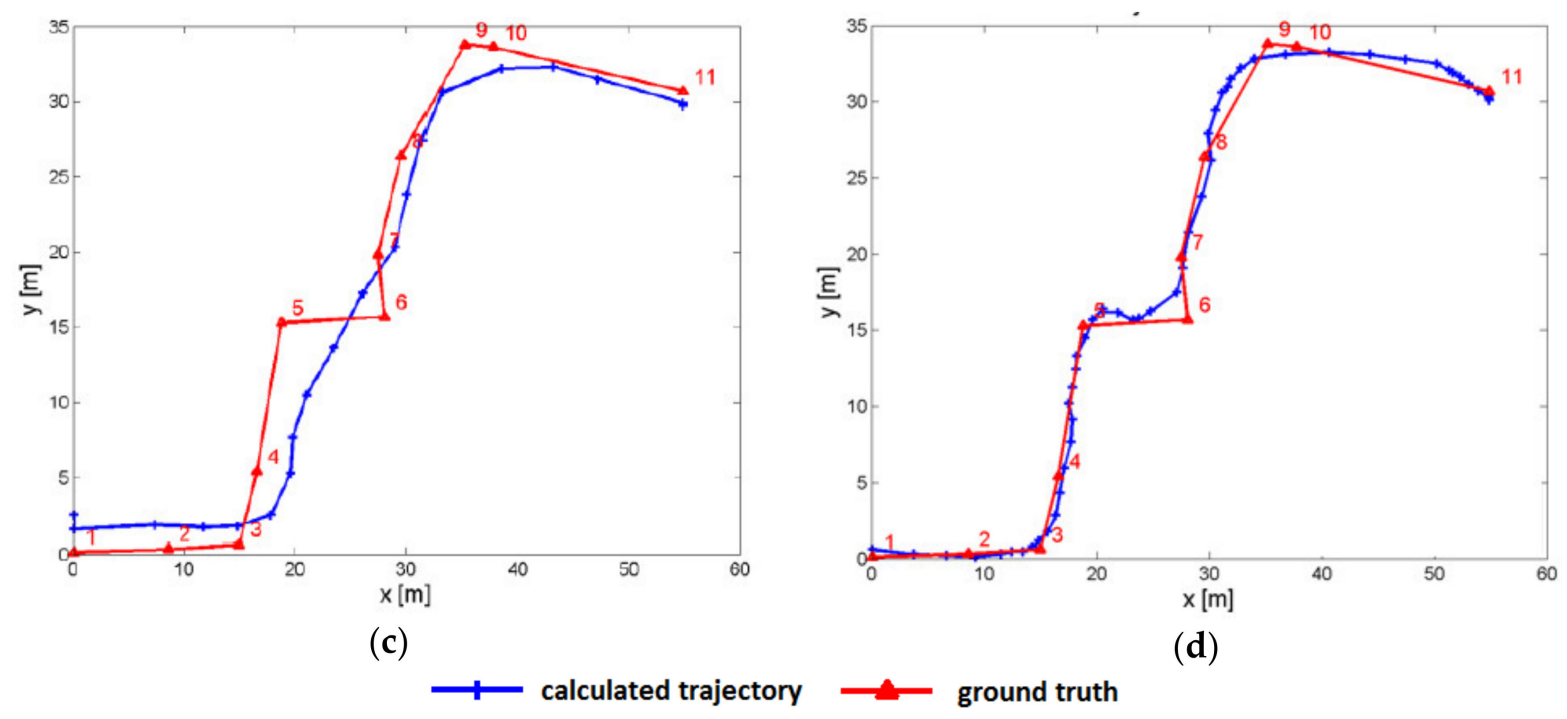
Resulting trajectories of a user walking along with usual step speed (two results for trajectory 1 (**a**,**b**) and two results for trajectory 2 (**c**,**d**)) with long scan duration of the smartphone (**a**,**c**) and short scan duration (**b**,**d**) resulting in significantly different numbers of trajectory waypoints.

The achieved positioning results of these two trajectories using fingerprinting are presented in Figure 16 (two results for trajectory 1 in Figure 16a,b and two results for trajectory 2 in Figure 16c,d. Figure 16a,c show the results with a smartphone with long scan duration and Figure 16b,d with short scan duration for each trajectory. This is why the results in Figure 16a,c for both trajectories show larger deviations from the ground truth, especially at the sharp turns. The average deviations range from 0.3 to 3.5 m with outliers showing maximum deviations of over 5 m. As discussed in Section 2.2.6, the scan duration has a major influence on kinematic positioning, as lesser observations are recorded if the scan duration is longer. Therefore, the overall best result was achieved along trajectory 2 with the smartphone, which took only around 1 s for each Wi-Fi scan.

### 4.3. Case Study 3: DWi-Fi

**Figure 17:**
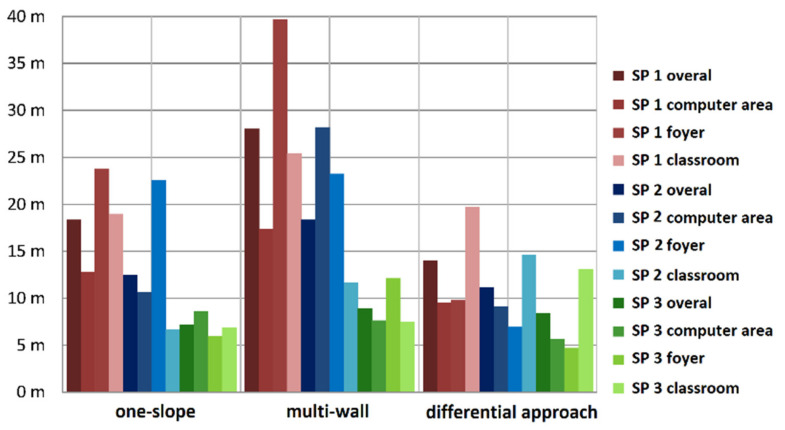
Comparison of the maximum deviations of the position estimates for kinematic positioning using the one-slope, multi-wall model, and differential approach (DWi-Fi) for three different smartphones (SP) in the different areas of the test site. The trajectory leads from the foyer through a classroom VII and an area with desktop computers (red trajectory in Figure 15a).

Figure 17 compares the results of kinematic RSS-based lateration using standard propagation models, i.e., the one-slop and multi-wall model (see Section 3.1.1) or the differential DWi-Fi algorithm (Section 3.2.3). Observations were carried out with three different smartphones in the same areas of the building, as shown in Figure 15a. As can be seen, DWi-Fi outperforms the standard models where the maximum deviations can reach tens of meters. Deviations are analyzed along the whole trajectory. Problems were here again the difficult environment and the device-dependent scan duration of the smartphones.

### 4.4. Case Study 4: Wi-Fi RTT

**Figure 18:**
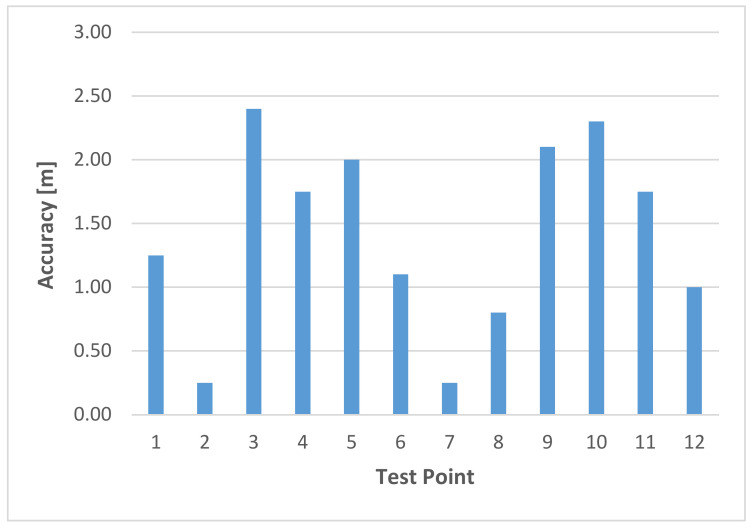
Comparison of the average deviations of the Wi-Fi RTT measurements on 12 test points evenly distributed in a grid with a spacing of 6 m between them.

The authors in [72] presented an evaluation and comparison study between Wi-Fi RTT and GPS-based localization in an outdoor space located in a central downtown area. These studies were based on the same testing environment and the same 12 test points distributed in a regular grid with spacing of 6 m between them. Both GNSS with the ranging code and Wi-Fi RTT observations with a smartphone were analyzed. Results showed that the average positioning accuracies from the two technologies are 5.1 m and 1.4 m on average, respectively. Thus, the Wi-Fi RTT technology demonstrated a much better performance both in accuracy and stability. Figure 18 presents the resulting average deviations from the ground truth on the 12 test locations. Moreover, the application of DWi-Fi could lead to further improvement.

### 4.5. Summary of the Major Findings

These examples demonstrate the difficulties that can arise in Wi-Fi smartphone positioning. Highly accurate and effective correction models and algorithms are required to achieve acceptable results. These are especially needed in kinematic positioning in real-world environments. In the case of RSS-based techniques, the use of standard signal propagation models mostly did not yield to the required performance level. No improvement in results was seen while using different modern smartphones with respect to the time needed for a single Wi-Fi scan. Thus, the presented results can be seen representative for a great number of mobile devices. The overall best performance is achievable if the positioning accuracy requirements are not set too high, such as at room-level granularity (see e.g., [12]). Thereby, different RSS-based lateration and fingerprinting approaches yielded similar performance levels in terms of positioning accuracy. However, fingerprinting can outperform RSS-based lateration in the case if the RSS values are very distinctive for certain APs in different areas of the environment, as the RSSI are used directly in the matching process. Especially, probabilistic fingerprinting algorithms can lead to better results; however, there is a tradeoff of higher workload in system training. The presented kinematic user localization in the case studies is much more challenging than static positioning. On the other hand, Wi-Fi RTT lateration may outperform RSS-based approaches under ideal conditions and in static scenarios. Thus, a combination with RSS-based techniques is a promising way to go. In addition, the application of DWi-Fi could lead to further improvement in the integration of both techniques.

## 5. Evolution of Wi-Fi Localization

In this section, selected Wi-Fi positioning systems are briefly discussed, demonstrating the evolution and maturation process of such systems. The brief system descriptions do not claim to cover all of them, as many researchers have worked on these developments. The overview just shows that there are a number of different approaches for positioning with Wi-Fi, which have different advantages and disadvantages.

One of the first Wi-Fi-based IPS is RADAR, which was developed by Microsoft Research [43]. It combines empirical fingerprinting measurements with a signal propagation model to determine the location of the user. Thereby, the propagation model takes into account a damping factor for the walls and the floor. The average accuracy obtained with the RADAR systems is in the range of 2 to 3 m.

COMPASS [73] is one of the first IPSs that takes into account the effect of user orientation. During the training phase, the Wi-Fi RSS are recorded in different orientations. Then, in the positioning phase, a digital compass is used to orientate the mobile device of its user. This information is used to minimize the influence of the human body of the user. A probabilistic approach is used to determine the position. The developers have shown that this system can achieve an average positioning accuracy of around 1.7 m under ideal conditions. A similar system is SMARTPOS [74], which is based on the deterministic fingerprinting method. A weighted *K*-nearest neighbor (WKNN) was applied achieving a mean positioning accuracy of at best 1.2 m.

Another IPS is Freeloc [75], where users collect Wi-Fi RSSs themselves. Due to this crowdsourcing method, the creation of the radio maps is done automatically, which saves a lot of time. One problem is device heterogeneity, as the signals are not measured to the same degree depending on their hardware. Therefore, in the Freeloc system, the absolute RSS values are not taken into account: only the relative signal strengths. Experiments conducted in several real-world environments showed that Freeloc delivers reliable results with similar accuracies as the systems described above.

Dari et al. [76] developed CAPTURE, an IPS based on fingerprinting that determines the position with the help of nearest neighbors (NN). However, the performance of the system is heavily influenced by the signal fluctuations and noise. WiDeep [77] is a deep-learning-based IPS that achieves high resolution and stable accuracy despite the presence of signal noise. The noise is minimized by means of an auto encoder. In addition, a number of additional components are installed to cope with overtraining and deal with heterogeneous smartphones. The results showed an average accuracy of 1.2 to 2.6 m.

Hou et al. [78] tested a Wi-Fi-based IPS by measuring the Angle-of-Arrival (AoA). The APs were equipped with antenna arrays and attached to the ceiling, so that the signals can reach the receiver directly. For localization, the surrounding APs return their location and direction angles that point to the users’ current position. The experimental results in a hospital showed that the positioning error in indoor environments is less than 2.5 m. However, the measurement of AoA is not very easy to realize and implement in practice.

Kulkarni and Lim [70] use the new Android Wi-Fi RTT technology to calculate the distance between APs and a smartphone. With the help of multi-lateration, a localization error of less than 1.5 m was achieved at the 95% reliability level. A comparison between GPS and Wi-Fi RTT-based localization using a smartphone as the end-user device outdoors in a relatively open urban area was conducted by Bai et al. [72]. The results showed that the average positioning accuracy for Wi-Fi RTT-based localization is 1.4 m (see case study 4 in Section 4.4), which was much better than the accuracy obtained from the GPS with the smartphone on the same test locations.

Guo et al. [68] combine RTT and RSS localization. The results show that this hybrid approach achieves a remarkable improvement in positioning accuracy, robustness, and update rate in both static and dynamic tests, including indoor and outdoor environments. Compared to standard fingerprinting, the performance of the IPS is significantly improved, and it achieves an average positioning accuracy of 1.4 m with an update rate of only about 0.2 s. Thus, the hybrid solution enhances kinematic user localization significantly.

Other research on Wi-Fi positioning focus on positioning algorithm improvement (see e.g., [51]), signal analysis [79,80], fingerprint database construction [80], and so on. Their fundamental purpose is to improve Wi-Fi positioning performance. Through analyzing the statistical properties of a large number of Wi-Fi signals, many researchers tried to analyze and explain the factors affecting indoor positioning accuracy and stability [22].

Huang et al. [80] presented a GraphSLAM-like algorithm for signal strength SLAM (Simultaneous Location and Mapping). The algorithm uses Gaussian process latent variable models (GP-LVM) and shares many of the benefits of Gaussian processes, yet it is viable for a broader range of environments, since it makes no signature uniqueness assumptions. This method works well in Wi-Fi signal-rich environments. Li et al. [12] considered a Hidden Markov Model (HMM) framework for probabilistic fingerprinting (see Section 3.1.4). The solution provides localization capabilities on a room-level or region-level granularity due to segmentation of the area of interest e.g., the building in cells. Such a segmentation will define the transition matrix in the HMM in such a way that only adjacent cells have non-zero transition probability, while the transition probability between isolated cells is zero. A Multivariate Gaussian Mixture Model (MVGMM) is fitted to model the probability distribution of RSS measurements in each cell to account for spatial correlation of the RSSs from multiple APs. Successful matching rates for mobile user tracking of over 98% were achieved for the allocation of the correct cell if a user walks along trajectories through rooms in a building [81].

The author of this contribution [15] introduced the aforementioned differential DWi-Fi approach for the first time (see Section 3.2.3), where reference stations are deployed, scanning and broadcasting Wi-Fi signals. A significant performance improvement in terms of robustness, reliability, and positioning accuracy is achieved due to the real-time modeling of signal fluctuations. DWi-Fi localization is applicable for both RSS and RTT lateration.

## 6. Conclusions and Outlook

The investigations conducted in the presented case studies have shown that Wi-Fi positioning can be used to achieve accuracies on the meter level and that the chosen novel directions are promising and highly suitable. Positioning by means of Wi-Fi thereby depends, among other things, on the fluctuations and noise of the signal and is normally not robust against dynamic changes in the environment. It was seen that a calibration with a multivariate linear regression model has to be carried out for each smartphone due to the device-dependent reception sensitivity. Using such a model, this device dependency could be reduced to a minimum and similar positioning accuracies for user localization in kinematic mode as for static and stop-and-go measurements obtained.

Conventional fingerprinting methods normally perform training using a site survey only once in the beginning and thereafter only if the environment changes significantly, which are not resistant to dynamic environment changes. Continuously updating the fingerprint database is a new way to enhance the achievable positioning accuracy. For this purpose, the continuous scanning of Wi-Fi signals is carried out to derive corrections for signal fluctuations. It is a network calibration method based on reference stations realized by low-cost computers, i.e., Raspberry Pi units, which are able to derive correction parameters in real-time. In this way, the measured RSS values at the user’s side are corrected, the fingerprinting database is continuously updated, and thus an adaption to the possible changes in the dynamics of the environment is achieved.

Novel developments for lateration based on measurement of the two-way travel times (RTT measurements with FTM) between the AP and the mobile client enhance the performance capabilities of Wi-Fi positioning in terms of robustness and achievable positioning accuracies even further. However, RSS-based techniques will continue to have their legitimacy in view of non-existent ubiquitous coverage with new hardware. Therefore, combinations will be most effective. Therefore, future work at TU Wien will continue on the work presented in the case studies and further development and enhancement of hybrid approaches to be able to benefit from the advantages of all available localization techniques. For this purpose, advanced algorithms and models have to be developed, tested, and analyzed.

## Figures and Tables

**Table 1:** IEEE 802.11 standard and its extensions.

	802.11	a	b	g	n		ac	ax	
alternative notation	-	-	-	-	Wi-Fi 4		Wi-Fi 5	Wi-Fi 6	
publishing year	1997	1999	1999	2003	2009		2013	2019	
frequency band [GHz]	2.4	5	2.4	2.4	2.4	5	5	2.4	5
bandwidth [MHz]	22	20	22	20	20, 40		20, 40, 80, 160	20, 40, 80, 160	
usable channels	13	19	13	13	13	19	19	8	
range [m]	20–100	35–120	40–140	40–140	250		50	N/A	

**Table 2:** Damping values for a signal frequency of 2.4 GHz [21].

Material	Damping [dB]
thin wall	2–5
brick wall	6–12
concrete wall	10–20
concrete ceiling	20–40
double glazing	25–35

**Table 3:** Received Signal Strengths (RSS) deviations in three orientations from the minimum fourth orientation.

	CP 13		CP 14	
	2.4 GHz	5 GHz	2.4 GHz	5 GHz
Orientation 1	4.8	8.1	-	-
Orientation 2	4.4	7.9	6.6	8.3
Orientation 3	-	-	6.8	8.1
Orientation 4	4.3	7.3	5.4	8.9
	4.5	7.7	6.3	8.4

**Table 4:** Parameter for the one-slope model [21].

	freq. [GHz]	*P*_0_ [dB]	γ [–]
office building	2.4	40.2	4.2
corridor	2.4	40.2	1.2
office building	5	46.8	4.6
